# Therapeutic landscape of ovarian cancer: recent advances and emerging therapies

**DOI:** 10.1186/s40364-025-00818-7

**Published:** 2025-08-12

**Authors:** Ling Wang, Qun Zhang, Xue Wang, Zixuan Dong, Shanshan Liu, Qi Wang, Zhiqiang Zhang, Junji Xing

**Affiliations:** 1 Department of Gynecology and Obstetrics, The Second Norman Bethune Hospital of Jilin University, 218 Ziqiang Street, Changchun, Jilin 130041 China; 2 Department of Gynecologic Oncology, First Ward, Jilin Provincial Cancer Hospital, 1018 Huguang Street, Changchun, Jilin 130022 China; 3 Immunobiology and Transplant Science Center, Department of Surgery, Houston Methodist Academic Institute, Houston Methodist, Houston, TX 77030 USA; 4 Department of Cardiovascular Sciences, Houston Methodist Academic Institute, Houston Methodist, Houston, TX 77030 USA; 5 Department of Surgery, Weill Cornell Medicine, Cornell University, New York, NY 10065 USA

**Keywords:** Ovarian cancer, PARP inhibitors, Antibody-drug conjugates (ADCs), Immunotherapy, Immune checkpoint inhibitors, Chimeric antigen receptor T (CAR-T) cell therapy, Tumor vaccines

## Abstract

Ovarian cancer ranks as the seventh most common malignancy and the eighth leading cause of cancer-related death in women worldwide. Most patients are diagnosed at an advanced stage, resulting in poor survival outcomes. The standard treatment is primary debulking surgery (PDS) with platinum-based chemotherapy, however interval debulking surgery (IDS) following neoadjuvant chemotherapy (NACT) is an alternative for select cases. In this review, we summarize recent advancements in the therapeutic landscape of ovarian cancer, focusing on targeted therapies, immunotherapy, and novel drug delivery systems. Poly (ADP-ribose) polymerase (PARP) inhibitors have markedly improved progression-free survival in BRCA-mutated and homologous recombination deficiency (HRD)-positive patients. Antibody-drug conjugates (ADCs), immune checkpoint inhibitors (ICIs), chimeric antigen receptor T (CAR-T) cell therapy, and tumor vaccines are emerging strategies, but they face challenges due to treatment resistance and tumor microenvironment suppression. Future research should focus on combination therapies, ADCs optimization, and immunotherapy refinement, while also integrating nanotechnology and 3D organoid models to enhance treatment precision to improve survival outcomes and quality of life for ovarian cancer patients.

## Introduction

**Fig. 1:**
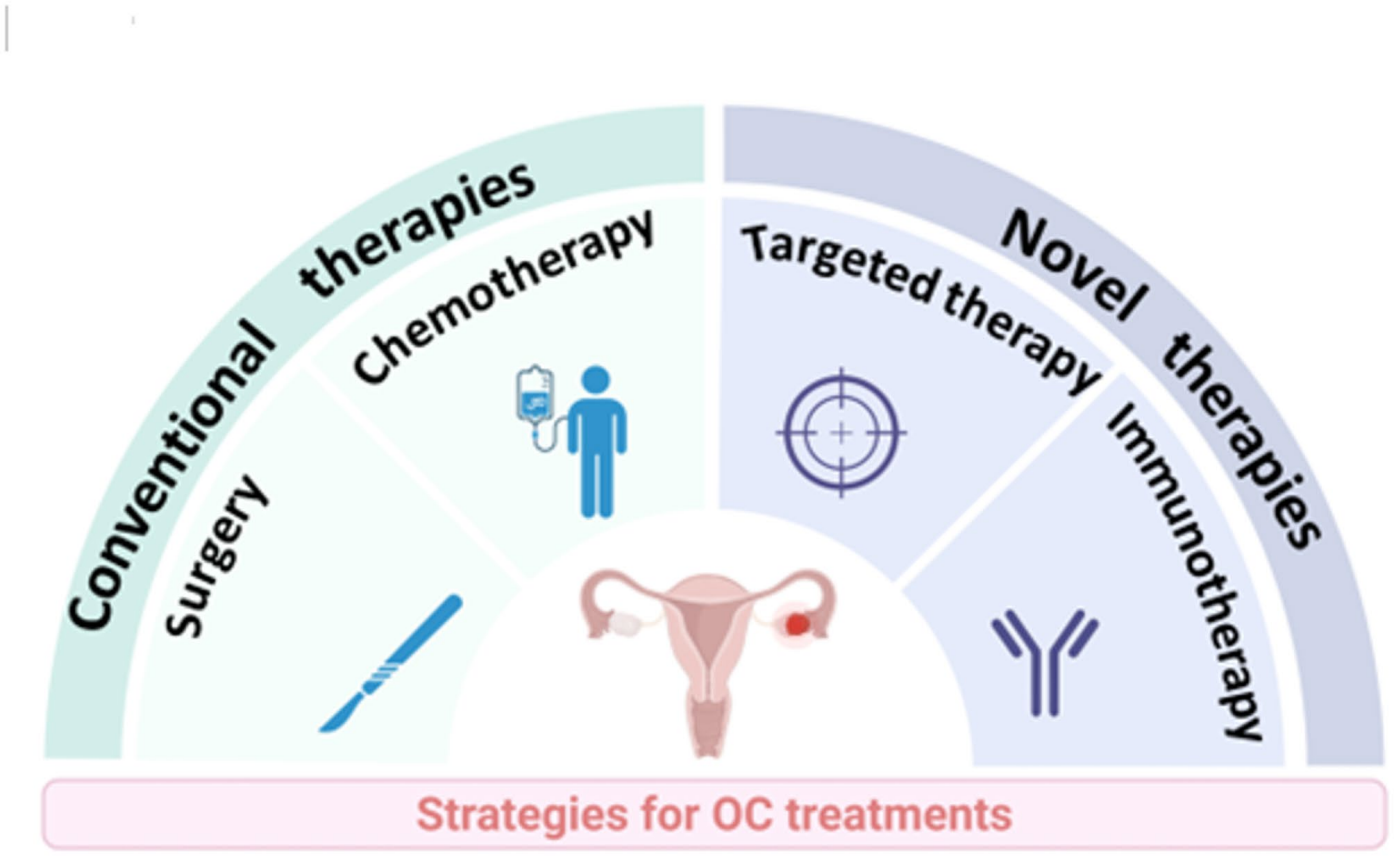
Strategies for OC treatments. The strategies for OC treatments encompass conventional therapies such as surgery and chemotherapy, novel targeted therapies including PARP inhibitors and antibody-drug conjugates (ADCs), and novel immunotherapies comprising immune checkpoint inhibitors (ICIs), Chimeric Antigen Receptor T (CAR-T) cell therapy, tumor vaccines, etc

Ovarian cancer (OC) is the seventh most common malignancy and the eighth leading cause of cancer-related mortality in women globally. In 2022, an estimated 324,398 new cases were diagnosed, resulting in 206,839 deaths worldwide, accounting for approximately 2.1% of all cancer-related deaths [1]. Epithelial ovarian cancer (EOC) constitutes 85%-90% of ovarian malignancies, with 75% of cases being diagnosed at an advanced stage (FIGO stage III/IV). Risk factors for EOC include inherited genetic mutations such as *BRCA1/BRCA2* as well as age, infertility, and nulliparity [2]. Currently, standard treatment approaches for OC comprise of conventional primary debulking surgery (PDS) combined with platinum-based chemotherapy or neoadjuvant chemotherapy (NACT) followed by interval debulking surgery (IDS) and postoperative platinum-based chemotherapy. In recent years, novel targeted therapies, notably PARP inhibitors and antibody-drug conjugates (ADCs), have ushered in a new era in OC management. Novel immunotherapies, including immune checkpoint inhibitors (ICIs), chimeric antigen receptor T (CAR-T) cell therapy and tumor vaccines, also represent critical advancements (Fig. 1).

## Surgery

### The efficacy of debulking surgery

Optimal cytoreductive surgery, which aims to remove all macroscopic tumor burden, is a key prognostic factor in AOC. A landmark randomized trial by Vergote et al. demonstrated that NACT followed by IDS is non-inferior to PDS in terms of progression-free survival (PFS) and overall survival (OS). Notably, complete macroscopic resection emerged as the strongest independent predictor of outcome [3]. Meta-analyses of the OVAR‑3, ‑5, and ‑7 trials, including 3,388 patients with FIGO stage II–IV EOC, showed that each 10% increase in tumor reduction is associated with a 5.5% improvement in median survival. In a pooled cohort of 3,126 patients, those with no residual disease post-surgery achieved a median survival of 99.1 months, compared to 36.2 months for ≤ 1 cm residual disease and 29.6 months for > 1 cm residual disease [4]. Reflecting these findings, the 2022 NCCN Ovarian Cancer Guidelines now mandate the complete removal of all visible pelvic, abdominal, and retroperitoneal disease as the primary surgical objective.

### The predictive value of scoring systems for optimal debulking surgery

To enhance the objective evaluation of tumor burden, peritoneal dissemination, and the likelihood of achieving complete cytoreduction, several scoring systems have been developed. These include the laparoscopic exploration scoring system (Fagotti score [5]), Fagotti modified score, CT-based score [6], Aletti score [7], complication score [8], peritoneal cancer index (PCI) score [9], Eisenkop score system [10] and other predictive models such as the R0 score [11]. This strategy has been shown to significantly improve patient prognosis (Table 1).

**Table 1:** Summary of studies on predictive scoring system for optimal cytoreduction

Author	Year	*N*	Stage	Study setting	Scoring system	Cut-off value	AUC	Criteria	PPV (%)	NPV (%)
Suidan [6]	2017	350	III–IV	prospective, non-randomized	PIV	3	0.72	CT + Clinical		
Fagotti [5]	2008	113	III-IV	prospective	PIV	8		Intraoperative findings	100	59.5
Jo´nsdo´ttir [9]	2020	167	III-IV	retrospective	PCI	24	0.95	Peritoneal tumor load	100	33
Chéreau [169]	2010	61	III-IV	retrospective	Eisonkop	10	0.63	Abdominal & pelvic tumor load	78	13
Feng [170]	2018	110	III-IV	prospective	Eisonkop		0.81	Peritoneal tumor load		
Dessapt [11]	2016	123	III-IV	prospective	R0	4	0.76	CT + PCI + Age		25.6
Aletti [7]	2010	576	III-IV	prospective	SCS	8				
Vizzielli G [8]	2016	555	III-IV	Prospective & retrospective	Complication Score system			complication		

## Chemotherapy

### Epithelial ovarian cancer (EOC)

Since the introduction of platinum-based chemotherapy in the 1970s, the paclitaxel-carboplatin (TC) regimen has been the cornerstone of first-line treatment for EOC, with bevacizumab providing additional benefits in select stage II–IV cases [12, 13]. Several trials have explored modifications to the standard TC regimen to improve efficacy and tolerability. The JGOG3016 trial reported significantly improved PFS and OS with a dose-dense TC regimen [14]. Conversely, the MITO-7 and ICON8 trials found no survival advantage with weekly or dose-dense schedules, though MITO-7 study noted improved quality of life and reduced hematologic toxicity with weekly dosing [15, 16]. Similarly, the SCOTROC trial showed comparable outcomes [17]. Collectively, these findings suggest that while alternative dosing strategies may enhance tolerability in certain patients, they do not consistently improve survival compared to the standard TC regimen.

### Hyperthermic intraperitoneal chemotherapy (HIPEC)

HIPEC gained clinical attention after Fagotti et al. reported on its combination with minimally invasive surgery in 2013 [18]. Subsequent studies in FIGO stages Ic-IIIc OC have suggested survival benefits with HIPEC [19]. The NCCN Guidelines endorse cisplatin-based HIPEC (100 mg/m²) during IDS for stage III OC in patients with response or stable disease after three cycles of NACT [20]. In contrast, the ESMO–ESGO consensus recommends restricting HIPEC use to well-designed randomized controlled trials, citing insufficient high-level evidence [21].

#### HIPEC in advanced OC (AOC)

Multiple randomized and cohort studies have evaluated the role of HIPEC in newly diagnosed AOC. A phase III randomized trial by Lim et al., involving interval cytoreductive surgery (ICS) following NACT, reported modest improvements in median PFS (17.4 vs. 15.4 months) and OS (61.8 vs. 48.2 months) with HIPEC [22]. In 2018, van Driel et al. showed that adding cisplatin (100 mg/m²) during HIPEC during IDS extended median OS from 33.9 to 45.7 months and recurrence-free survival (RFS) from 10.7 to 14.2 months, without increased severe toxicity [23]. The OVHIPEC (NCT00426257) phase III trial confirmed significant improvements in both RFS and OS with HIPEC in patients initially deemed inoperable [24], supporting the findings of Lim and van Driel. Collectively, current evidence endorses cisplatin-based HIPEC during interval surgery to significantly enhance survival in AOC, with emerging biomarker data providing mechanistic support.

#### HIPEC in recurrent OC (ROC)

The role of HIPEC in ROC remains controversial. Studies by Fagotti and Safra et al. reported improved PFS and OS, particularly in platinum-sensitive and Breast Cancer susceptibility gene (BRCA)-mutated patients, when HIPEC is combined with secondary cytoreductive surgery (CRS) [25, 26]. Conversely, trials by Bakrin and Baiocchi et al. found no significant survival benefit [27, 28]. Furthermore, HIPEC is associated with increased grade III–IV morbidity. Notably, the GOG-0213 trial showed no OS advantage with secondary CRS alone [29]. Ongoing randomized trials (HORSE, CHIPOR) are expected to clarify HIPEC’s therapeutic value in this setting [30, 31].

#### Pressurized intraperitoneal aerosol chemotherapy (PIPAC)

PIPAC is an innovative drug delivery approach for patients with peritoneal metastases, enhancing intraperitoneal drug distribution and tissue penetration. Clinical studies have confirmed its feasibility, safety, and tolerability, with low rates of manageable adverse events [32]. In OC, PIPAC has shown objective response rates (ORR) of 62–88% and median survival of 11–14 months, while maintaining quality of life even with repeated administration [33]. Despite these promising results, PIPAC remains investigational, requiring further validation through well-designed prospective trials before widespread clinical adoption.

## Poly (ADP-ribose) polymerase (PARP) inhibitors

**Fig. 2:**
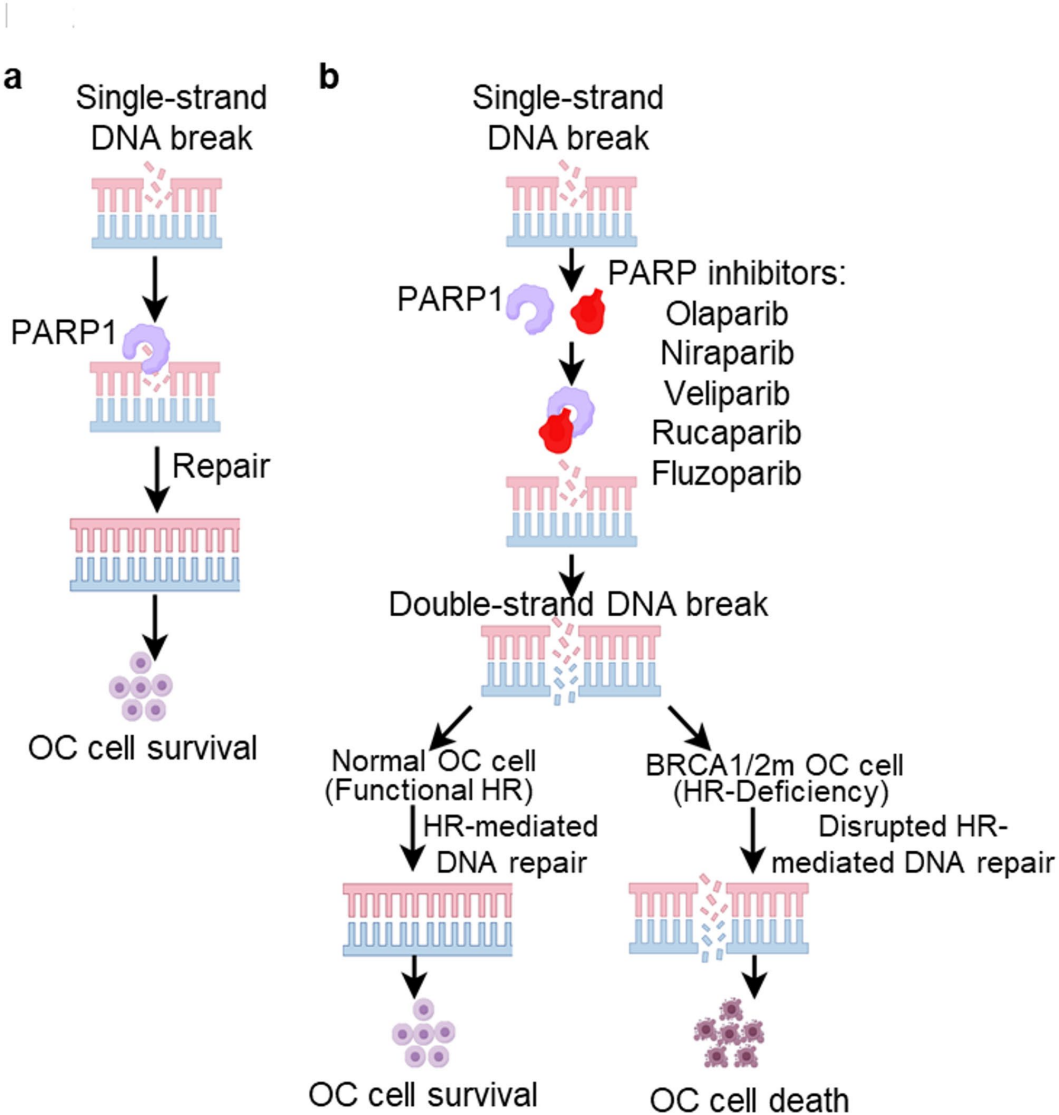
Mechanism of targeted therapy using PARP inhibitors for OC treatment. (**a**) Endogenous single-strand DNA breaks (SSBs) frequently occur in rapidly proliferating cancer cells, including OC cells. PARP1 plays a crucial role in repairing SSBs, which is essential for OC cell survival. (**b**) PARP inhibitors, such as Olaparib, Niraparib, Veliparib, Rucaparib, and Fluzoparib, block PARP1 binding to DNA breaks, preventing SSB repair. Unrepaired SSBs can degenerate into double-strand DNA breaks (DSBs) that are toxic to cells. Homologous recombination (HR) is the primary repair mechanism for DSBs during cell replication. In normal OC cells with functional HR, SSB-derived DSBs are repaired, ensuring genome stability and OC cell survival. However, in BRCA1/2-mutated (BRCA1/2m) OC cells with HR-deficiency, DSBs remain unrepaired, leading to OC cell death

PARP inhibitors (PARPi), including olaparib, niraparib, rucaparib, veliparib, and fluzoparib, exert their antitumor effects by blocking the repair of endogenous single-strand DNA breaks (SSBs), leading to replication-associated double-strand breaks (DSBs) [34]. In homologous recombination (HR)-deficient OC cells, such as those with BRCA1/2 mutations, these DSBs cannot be efficiently repaired, resulting in cell death. This synthetic lethality forms the mechanistic basis for PARPi efficacy in HR-deficient OC tumors (Fig. 2).

Recent phase III trials (SOLO1/SOLO2, PRIMA, PAOLA‑1, ATHENA‑Mono, ARIEL3) have shown significant PFS benefits with PARPi maintenance therapy, especially in patients with BRCA mutations or HR deficiency (HRD) [35]. Notably, olaparib is the only PARPi with mature OS data, while niraparib and rucaparib have gained broad approvals, including in non‑BRCA and HR-proficient (HRP) settings.

### Olaparib

#### FDA approvals and guidelines recommendations

Olaparib has received progressive FDA approvals based on robust clinical evidence. In 2014, it was approved for AOC in patients with deleterious or suspected deleterious germline BRCA (gBRCA) mutations after ≥ 3 prior lines of chemotherapy. In 2017, olaparib received full approval for maintenance therapy in ROC following response to platinum-based chemotherapy, supported by the Study 19 and SOLO2 trials [36, 37]. The SOLO-1 trial led to a 2018 approval for first-line maintenance therapy in BRCA-mutated advanced EOC patients achieving complete response (CR) or partial response (PR) after platinum-based treatment, showing significant improvements in PFS [38–40]. In 2020, the PAOLA-1 trial supported approval of olaparib combined with bevacizumab for HRD-positive advanced EOC as first-line maintenance therapy [41, 42]. However, in 2023, this indication was narrowed to exclude BRCA wildtype patients, and in 2024, the original 2014 accelerated approval for the capsule formulation (≥ 3 prior lines in gBRCA-mutated patients) was withdrawn, as the tablet formulation become standard [43–45]. Collectively, these regulatory decisions highlight olaparib’s therapeutic value in BRCA-mutated and HRD-positive OC, particularly for maintenance therapy (Table 2).

**Table 2:** Clinical trials evaluating maintenance therapy with olaparib

Clinical trial	Experimental arm	Control arm	regimen	patients	PFS, mo	PFS HR	OS, mo	OS HR
Study19 [37] NCT00753545	olaparib	placebo	400 mg, PO, Bid	Total:265 133 vs. 132	8.4 vs. 4.8	0.35 (95% CI: 0.25–0.49; *p* < 0.0001)	29.8 vs. 27.6	0.73 (95% CI, 0.55–0.95; *P* = 0.0238)
Solo2 [36] NCT01874353	olaparib	placebo	300 mg, PO, Bid	Recurrent EOC gBRCAm Total: 295 196 vs. 99	19.1 vs. 5.5	0.30 (95% CI, 0.22–0.41; *P* < 0.0001)	51.7 vs. 38.8	0.74 (95% CI, 0.54–1.00; *P* = 0.054)
Solo1/GOG3004 [40] NCT01844986	olaparib	placebo	300 mg, PO, Bid	Newly diagnosed EOC BRCAm 260 vs. 131	64 vs. 15.1	0.37 (95% CI, 0.28–0.48;	alive 67% vs. 46.5%	
Solo3 [43] NCT02282020	Olaparib	Nonplantinum chemotherapy	300 mg, PO, Bid	gBRCAm PSR SOC Total:266 178 vs. 88	13.4 vs. 9.2	0.62(95% CI, 0.43–0.91)	34.9 vs. 32.9	1.07(95% CI, 0.76–1.49)
OReO [171] NCT03106987	olaparib	placebo	Olaparib 300 mg PO BID	Recurrent, platinum-sensitive EOC with exposure to prior PARPi	BRCAm 4.3 vs. 2.8 NonBRCAm 5.3 vs. 2.8	0.57(95% CI, 0.37–0.87; *P* = 0.022) 0.43(95% CI, 0.26–0.71; *P* = 0.0023)		
OPINION [172] NCT03402841 Single arm	olaparib		300 mg PO BID	PSR OC Non-gBRCAm	Total 9.2 sBRCAm 16.4 HRD + including sBRCAm 11.1 HRD + excluding sBRCAm 9.7 HRD- 7.3			
PAOLA-1/ ENGOT-ov25 [173] NCT2477644	Olaparib + bevacizumab	placebo + bevacizumab	300 mg, PO, Bid + bevacizumab 15 mg/kg every 3 weeks	Newly diagnosed EOC HRD+ 806 Higer- risk 595	37.2 vs. 17.7	0.33; 95% CI: 0.25–0.45	65.5% vs. 48.4%	0.62 (95% CI 0.45–0.85)
ORZORA [174] NCT02476968 Single arm	Olaparib		400 mg, PO, Bid	PSROC BRCAm or Non BRCA HRRm	BRCAm 18.0 sBRCAm 16.6 gBRCAm 19.3 Non-BRCA HRRm 16.4	95% CI: 14.3–22.1 95% CI: 12.4–22.2 95% CI: 14.3–27.6 95% CI: 10.9–19.3	BRCAm 46.8 sBRCAm 43.2 gBRCAm 47.4 Non-BRCA HRRm 44.9	95% CI: 37.9–54.4 95% CI: 31.7-NC 95% CI: 37.9-NC 95% CI: 28.9-NC

The NCCN and ESMO guidelines recommend olaparib as first-line maintenance therapy in patients with stage II–IV high-grade EOC harboring deleterious or suspected deleterious gBRCA1/2 or somatic BRCA1/2 (sBRCA1/2) mutations. Additionally, olaparib is endorsed for platinum-sensitive recurrent (PSR) EOC following CR or PR to platinum-based chemotherapy, particularly in BRCA-mutated tumors. For HRD-positive advanced EOC, olaparib combined with bevacizumab is recommended as first-line maintenance therapy after response to platinum-based treatment. These guidelines emphasize olaparib’s pivotal role in personalized management of BRCA-mutated and HRD-positive OC.

#### Emerging clinical trials exploring novel combination therapies

In the phase II BOLD trial, GINECO investigators evaluated a triplet regimen of olaparib, bevacizumab, and durvalumab in platinum-resistant recurrent (PRR) and PSR OC. The trial reported a 3‑month non‑progression rate of 69.8% (90% CI 55.9–80.0%) in PRR OC and a 6‑month non‑progression rate of 43.8% (90% CI 29.0–57.4%) in PSR OC, with median PFS of 4.1 and 4.9 months, respectively [46]. The phase III DUO‑O study (NCT03737643) showed a significant PFS benefit when durvalumab was added to chemotherapy and bevacizumab, followed by maintenance with olaparib and bevacizumab (median PFS: 25.1 vs. 19.3 months; HR 0.61) [47]. Conversely, the ICON9 trial (NCT03278717) reported no significant improvement in PFS or OS with olaparib plus cediranib compared to olaparib alone [48].

### Niraparib

#### FDA approval and guidelines recommendations

Niraparib received FDA approval in 2017 for maintenance therapy in ROC following response to platinum-based chemotherapy, based on the phase III NOVA trial [49]. In 2019, its indication expanded to include HRD-positive AOC after three or more prior chemotherapy lines [50]. In 2020, niraparib was approved for first-line maintenance therapy in AOC, regardless of biomarker status [51]. However, in 2022, updated OS data led the FDA to restrict its second-line maintenance indication to ROC patients with gBRCA mutations [52].

The NCCN and ESMO guidelines recommend niraparib for multiple OC settings: First-line maintenance therapy for advanced EOC with CR or PR to platinum-based chemotherapy, irrespective of BRCA mutation or HRD status (1); Second-line maintenance therapy for ROC with deleterious or suspected deleterious gBRCA mutations (2); Late-line monotherapy for HRD-positive AOC after three or more prior chemotherapy lines.

#### Clinical trials of niraparib maintenance therapy

The PRIMA trial showed improved 5-year PFS with niraparib maintenance, particularly in the HRD subgroup, with OS data evaluated at 60% maturity [51, 53]. The international AGO-OVAR 28/ENGOT-ov57 phase III study is comparing niraparib maintenance alone versus in combination with bevacizumab following chemotherapy, with primary endpoint results expected by 2028 [54, 55]. Meanwhile, the ongoing SOC-3 trial is evaluating whether complete secondary cytoreductive surgery (CRS) followed by niraparib maintenance provides additional survival benefits over chemotherapy and maintenance alone in patients with secondary ROC [56].

Several clinical trials have evaluated niraparib maintenance therapy across various settings: NOVA, NORA, NSGO-ANANOVA2, and ANITA trials for PSR-OC [57]; PRIMA, PRIME, and OVARIO trials for first-line maintenance [58]; the ongoing ANNIE trial for last-line treatment [59]. A comprehensive summary is provided in Table 3.

**Table 3:** Clinical trials evaluating maintenance therapy with nirparib

Clinical trial	Experimental arm	Control arm	regimen	patients	PFS, mo	PFS HR	OS, mo	OS HR
PRIMA [51] NCT02655016	Niraparib	placebo	300 mg, PO, Qd	Total:733 487 vs. 246 HRd: 373 247 vs. 126 HRp: 249 169 vs. 80	13.8 vs. 8.2 24.5 vs. 11.2 8.4 vs. 5.4	0.66 (95% CI: 0.55–0.78) 0.51 (95% CI: 0.40–0.66) 0.67 (95% CI: 0.50–0.89)	46.6 vs. 48.8 71.9 vs. 69.8 36.6 vs. 32.2	1.01 (95% CI, 0.84–1.23) 0.95 (95% CI, 0.70–1.29) 0.93 (95% CI, 0.69–1.26)
NOVA [175] NCT01847274	Niraparib	AS	300 mg, PO, Qd	BRCAwt ROC Total: 906 199 vs. 707 NOVA study-like population 123 vs. 143			24.1 vs. 18.4 28.1 vs. 21.5	0.8 (95% CI, 0.7–0.9) 0.6 (95% CI, 0.5–0.9)
NORA [52] NCT03705156	Niraparib	placebo	200/300 mg, PO, Qd	PSROC Overall population 177 vs. 88 gBRCAm 65 vs. 35 HR 112 vs. 53			51.5 vs. 47.6 56 vs. 47.6 46.5 vs. 46.9	0.86 (95% CI, 0.6–1.23) 0.86 (95% CI, 0.46–1.58) 0.87 (95% CI, 0.56–1.35)
NSGO-AVANOVA2/ENGOT-ov24 NCT02354131 [55]	Niraparib + bevacizumab 300 mg, PO, Qd+ 15 mg/kg every 3weeks	Niraparib 300 mg, PO, Qd		PSR OC Total:97 48 vs. 49	11.9 vs. 5.5	0.35(95% CI, 0.21–0.57)		
PRIME [176] NCT0370931	Niraparib	placebo	200/300 mg PO Qd	New diagnosed OC 255 vs. 129	24.8 vs. 8.3 NonBRCAm 5.3 vs. 2.8	0.45(95% CI, 0.34–0.60; *P* < 0.001) 0.43(95% CI, 0.26–0.71; *P* = 0.0023)		
QUADRA [50] NCT02354586 Single arm	Niraparib		300 mg PO Qd	ROC Total: 463	HRD + PSROC 5.5	(95% CI, 3.5–8.2)	17.2	(95% CI, 14.9–19.8)
OVARIO [58] NCT03326193 Single arm	Niraparib + bevacizumab		300 mg, PO, Qd+ bevacizumab 15 mg/kg every 3 weeks	Newly diagnosed EOC Total: 105	HRD+ 28.3 HRD- 14.2 HRD unkwon 12.1	95% CI: 19.9-NC 95% CI: 8.6–16.8 95% CI: 8.0-NC		
ANNIE [59] NCT04376073 Single arm	Niraparib + anlotinib		Niraparib 200/300 mg, PO, Qd + anlotinib 10 mg PO Qd (D1-D14) Q3 wk	PR ROC Total: 40	9.2	95% CI: 7.4–11.9	15.3	13.9-not evaluable
ANITA [57] (NCT03598270)	Niraparib + atezolizumab	Niraparib + placebo	Niraparib 200/300 mg, PO, Qd + atezolizumab 1200 mg IV (D1) Q3 wk	ROC Total: 417	11.2 vs. 10.1	0.89 (95% CI: 0.71–1.10; *P* = 0.28)		

### Other PARP inhibitors

#### Veliparib

Clinical trials have demonstrated the potential of veliparib in treating OC, particularly in patients with BRCA mutations. In a phase III trial, Coleman et al. evaluated veliparib both as a first-line therapy and maintenance treatment for OC [60]. Their findings showed that veliparib, when combined with chemotherapy, significantly improved PFS compared to chemotherapy alone, supporting its role in the management of AOC [61].

#### Rucaparib

Rucaparib has shown robust efficacy in BRCA-mutated OC, with early-phase trials demonstrating durable responses in patients with gBRCA mutations [62]. The ARIEL3 phase III trial reported significantly prolonged PFS with rucaparib as maintenance therapy for PSR OC [63]. The ARIEL4 study further confirmed rucaparib’s superiority over chemotherapy in extending PFS in relapsed BRCA1/2-mutated OC, though no OS benefit was observed [64], likely due to high crossover rates and BRCA reversion mutations. Both ESMO/ESGO and ASCO guidelines recommend rucaparib as maintenance therapy for patients with PSR OC, particularly those with BRCA mutations or HRD. Additionally, ESMO endorses rucaparib monotherapy for relapsed BRCA-mutated OC in patients intolerant to further platinum-based chemotherapy.

#### Fluzoparib

Fluzoparib was evaluated in the randomized, double-blind, placebo-controlled phase III FZOCUS-2 trial, which demonstrated significant PFS improvement (median 12.9 vs. 5.5 months; HR 0.25; *P* < 0.0001) as maintenance therapy in PSR OC, including patients with gBRCA mutations [65]. A prior phase II study in heavily pretreated gBRCA1/2mutant PSR OC reported an objective response rate (ORR) of approximately 70% and a median PFS of nearly 12 months, with a welltolerated safety profile [66].

## Antibody-drug conjugates (ADCs)

**Fig. 3:**
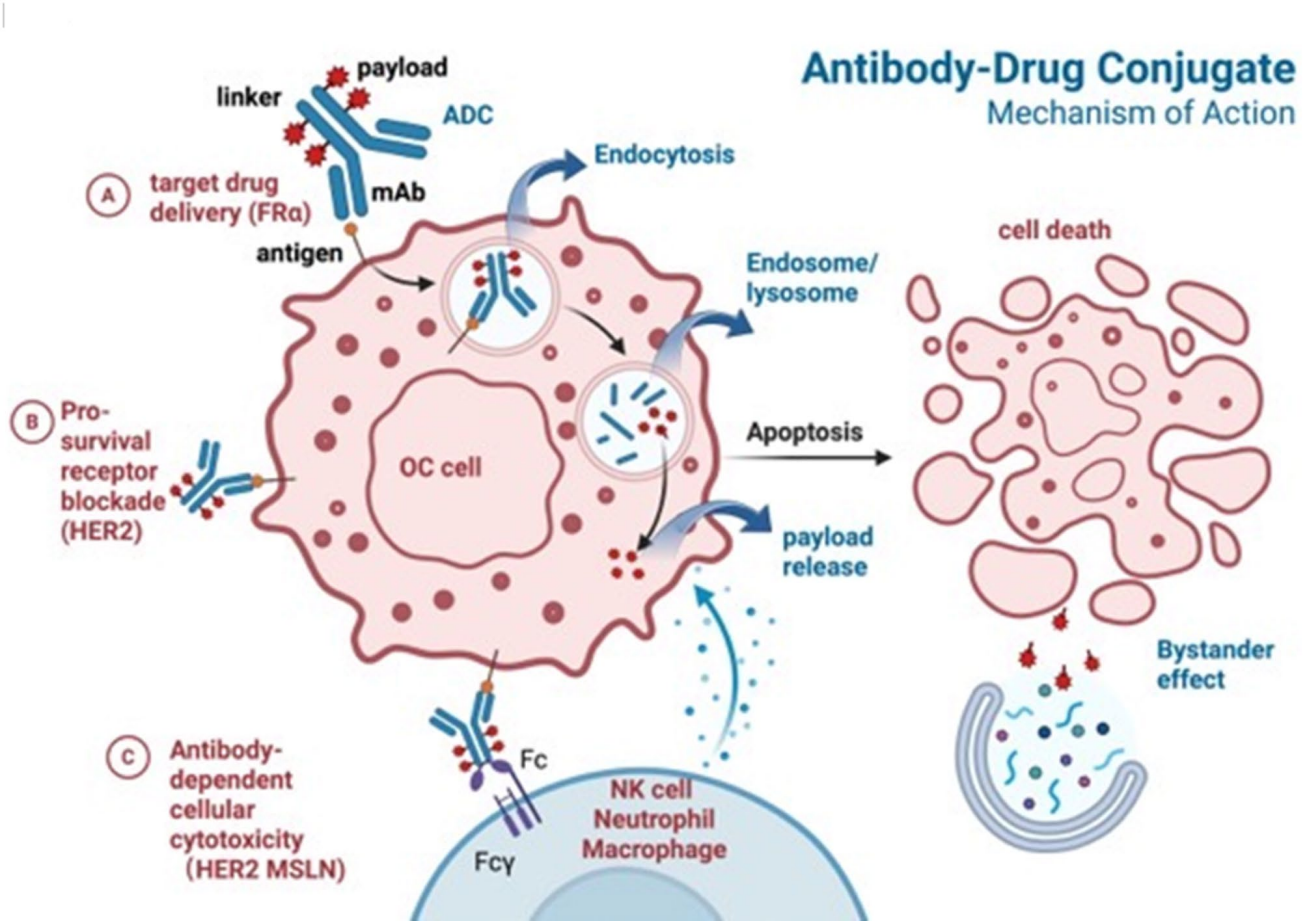
Mechanism of ADCs for OC treatment. Antibody-drug conjugates (ADCs) consist of an antibody bound to a cytotoxic drug through a linker. The antibody targets specific cancer cell antigens, binding to them and entering the cell via receptor-mediated endocytosis. Once inside, the cytotoxic drug is released, typically through enzymatic cleavage, and it kills the OC cell by disrupting essential processes like DNA replication, microtubule disruption, and immune modulation, among other mechanisms. This targeted approach allows for the delivery of potent chemotherapy specifically to OC tumor cells with minimum effect on normal tissues

Antibody-drug conjugates (ADCs) are a promising therapeutic class for OC, delivering cytotoxic payloads via targeted antibodies to improve outcomes in treatment-refractory disease, particularly platinum-resistant OC (PROC). ADCs targeting folate receptor alpha (FRα) and human epidermal growth factor receptor 2 (HER2) have shown significant potential [67]. Ongoing development has expanded ADC pipelines to target a wider range of antigens, including sodium-dependent phosphate transporter 2B (NaPi2b), mesothelin (MSLN), dipeptidase 3 (DPEP3), tissue factor (TF), and cadherin-6 (CDH6), enhancing the potential of ADCs as targeted therapies for OC [68] (Fig. 3; Tables 4 and 5).

**Table 4:** Clinical trials of ADCs in ovarian cancer

Antigens	Studies	Experimental arm	Control arm	Study detail	Patients (Number)	ORR	DoR (month)	PFS (month)	OS (month)
FRα	FORWARD I [69] NCT02631876	Mirvetuximab Soravtansine (MIRV)	Chemotherapy IC	phase III	FRα^**+**^aEOC Total:366 243 vs. 123	High FRα subgroup 24% vs. 10%		4.1 vs. 4.4 HR = 0.98 (95% CI, 0.73–1.31; *P* = 0.897)	
	SORAYA [69] NCT04296890	MIRV		Phase III	High FRα^**+**^ PROC aHGSOC *N* = 106	32.4%	6.9	4.27	15 (95% CI, 11.5–18.7)
	MIRASOL [70] NCT04209855	MIRV	Chemotherapy IC	Phase III	High FRα^**+**^ PROC aHGEOC Total:453 227 vs. 226	42% vs. 16% HR = 3.81 *P* < 0.001		5.62 vs. 3.98 HR = 0.65 *P* < 0.001	16.4 vs. 12.7 HR = 0.67 *P* = 0.0046
	FORWARD II [71] NCT02606305	MIRV + Bev		phase Ib/II	FRα^**+**^aEOC *N* = 94	44%	9.7	8.2	
	PICCOLO [73] NCT05041257	MIRV		phase II	High FRα^**+**^ PSROC *N* = 79	51.9% (95% CI:40.4–63.3)	8.25 (95% CI: 5.55–10.78)	6.93(95% CI: 5.85–9.59)	27.2(95% CI: 23.8–not reach)
	STRO-002 [77] NCT03748186	Luveltamab tazevibulin		phase I/II	PROC *N* = 33	33–47%			
HER2	DESTINY- PanTumor02 NCT04482309 [82]	T-DXd		phase II	HER2+ *N* = 267 HER2 2+/3+ *N* = 75	overall: 37.1%(95% CI:31.3–43.2) HER2 2+/3+ 61.3%(95% CI:49.4–72.4)	overall: 11.3(95% CI:9.6–17.8) HER2 2+/3+ 22.1(95% CI:9.6-not reach)	overall: 6.9(95% CI:5.6-8.0) HER2 2+/3+ 11.9(95% CI:8.2–13.0)	overall: 13.4(95% CI:11.9–15.5) HER2 2+/3+ 21.1(95% CI:15.3–29.6)
NaPi2b	NCT01363947 [86]	LIFA		phase I	PROC *N* = 24	46%			
	NCT01995188 [87]	LIFA with carboplatin ± bevacizumab		Ib	PSOC OR NSCLC	59%		10.7(95% CI:8.54–13.86)	
	NCT01991210 [88]	LIFA	PLD	phase II	PROC *N* = 95	34% vs. 15% (*P* = 0.03)		5.3 vs. 3.1 HR = 0.78 (95% CI, 0.46–1.31; *P* = 0.34)	
	NCT03319628 [89]	UpRi		phase I	PROC NSCLC *N* = 38	34%	5		
	UPLIFT [89]	UpRi		phase II	PROC NSCLC *N* = 268	Overall 13.1% (95% CI: 9.3–17.7%) NaPi2b-positive subgroup 15.6%(95% CI: 10.0-22.7%)	7.4		
MSLN	NCT02751918 [91]	AR + PLD		phase Ib	PROC *n* = 65	27.7%	7.6	5.0	
	NCT03587311 [92]	AR + Bev	Paclitaxel + Bev	phase II	PROC	21% vs. 65%		5.3 vs. 12.7 HR 2.02, *p* = 0.03	
	NCT01469793 [93]	DMOT4039A		phase I	PROC *N* = 31	13%		4.9-5.0	
DPEP3	NCT02539719 [94]	Tamrintamab pamozirine (SC-003)		phase I	DPEP3-positive OC *N* = 74	4%			
TF	InnovaTV 201 NCT02001623 [95]	TV		phase I/II	Solid tumors *N* = 147	15.6% (95% CI: 10.2–22.5%)			

**Table 5:** Ongoing clinical trials of ADCs in ovarian cancer stratified by target and treatment setting

Trial Name (NCT No.)	Phase	ADCs	Target	Population	Treatment setting	Key endpoints
GLORIOSA (NCT05445778)	Phase III	Mirvetuximab Soravtansine (MIRV)+Bev	FRα	FRα-high PSOC (≥ 75% cells, 2 + IHC) after platinum	Maintenance post-platinum	PFS
RAINFOL (NCT06619236)	Phase III	Rinatabart Sesutecan (Rina-S)	FRα	HGSOC/endometrioid OC, post 1–4 lines	Recurrent/progressive	PFS, OS, safety, QoL
REFRaME-O1 (NCT05870748)	Phase II/III	Luveltamab Tazevibulin (STRO-002)	FRα	FRα-positive PROC	Recurrent	ORR, DCR, safety
IMGN853-0420 (NCT05456685)	Phase II	MIRV + Carboplatin	FRα	FRα-positive PSOC post-first-line relapse	Combination + Maintenance	PFS, safety
NCT04606914	Phase II	MIRV + Carboplatin	FRα	Newly diagnosed advanced FRα-high OC	Neoadjuvant	Surgical outcome, ORR
GOG-3115 (NCT06890338)	Phase II	MIRV + Carboplatin (± Bev)	FRα	FIGO III–IV FRα-high OC	Neoadjuvant	ORR, PFS, surgical outcome
UP-NEXT (NCT05329545)	Phase III	Upifitamab Rilsodotin (UpRi)	NaPi2b	Recurrent PSOC post platinum-based therapy	Maintenance	PFS, OS
REJOICE-Ovarian01 (NCT06161025)	Phase II/III	Raludotatug Deruxtecan (R-DXd)	CDH6	PROC, HGSOC or endometrioid OC (1–3 prior lines)	Recurrent	ORR, PFS, CDH6 stratification

### ADCs targeting FRα

#### Mirvetuximab soravtansine (MIRV)

MIRV, an FRα-targeted ADC, has shown significant efficacy in FRα-high PROC, defined as ≥ 75% tumor cells with ≥ 2 + immunohistochemistry (IHC) staining. In November 2022, MIRV received accelerated FDA approval for FRα-high PROC following 1–3 prior lines of therapy, based on the SORAYA trial (NCT04296890), which reported a confirmed ORR of 32.4% and duration of response (DoR) of 6.9 months in a biomarker-selected population [69]. The confirmatory MIRASOL trial (NCT04209855) met its primary endpoint of PFS, establishing MIRV as the first ADC approved for OC [70].

Combination strategies with bevacizumab (FORWARD II trial) [71] and rucaparib (NCT03552471) [72], as well as monotherapy in heavily pretreated platinum-sensitive OC (PSOC) or PROC after ≥ 3 prior therapies (PICCOLO trial) [73], achieved ORRs ranging from 43 to 52% and median PFS ranging from 6.6 to 8.2 months. Toxicities, primarily gastrointestinal and ocular, were manageable. However, challenges such as FRα heterogeneity and limited bystander effect from MIRV’s DM4 payload highlight the need for biomarker-driven patient selection and rational combination therapies to address resistance [74].

Ongoing trials include the phase III GLORIOSA trial (NCT05445778), evaluating MIRV plus bevacizumab versus bevacizumab alone as maintenance therapy in FRα-high PSOC [75, 76]. The IMGN853-0420 trial (NCT05456685) [70] and GOG-3115 (NCT06890338) are investigating MIRV with carboplatin in relapsed and neoadjuvant settings, respectively, while NCT04606914 explores its neoadjuvant use in newly diagnosed advanced-stage FRα-high OC [72].

#### Luveltamab tazevibulin (STRO-002)

STRO-002, a novel FRα-targeted ADC, comprises a high-affinity anti-FRα antibody, a cleavable SC239 linker, and a potent tubulin inhibitor payload (SC209). Developed via a cell-free synthesis platform, it demonstrates activity in multidrug-resistant OC, with bystander killing in heterogeneous FRα-expressing models. The phase I/II STRO-002-GM1 trial (NCT03748186) reported ORRs up to 43.8% in PROC patients with high FRα expression and a manageable safety profile. Neutropenia, the primary treatment-related adverse event, was effectively mitigated with G-CSF support; ocular and pulmonary toxicities were minimal [77].

The ongoing REFRaME-O1 trial (NCT05870748) evaluates STRO-002 in FRα-positive PROC, including low-to-intermediate expression levels. Interim data show a 32% ORR and 96% disease control rate (DCR) across a broad range of FRα expression, with manageable hematologic toxicity and no interstitial lung disease [78].

#### Rinatabart sesutecan (Rina-S)

The phase III RAINFOL trial (NCT06619236, GOG-3107) is evaluating Rina-S versus chemotherapy in recurrent OC with high and low FRα expression. Prior phase I/II data showed a 50% ORR, even in low or absent FRα expression, suggesting broader clinical utility for FRα-targeted therapy beyond conventional expression thresholds [79, 80].

### ADCs targeting HER2

**Trastuzumab deruxtecan (T-DXd)**, a HER2-targeted ADC, combines a humanized trastuzumab backbone with a membrane-permeable topoisomerase I inhibitor via a cleavable peptide linker [81]. Although HER2 overexpression occurs in only 10–20% of OC cases, primarily in clear cell and high-grade serous OC (HGSOC), T-DXd has shown promising activity in this setting [82]. In the phase II DESTINY-PanTumor02 trial (NCT04482309), patients with HER2-expressing OC achieved an ORR of 63.6% in those with IHC 3 + tumors [83]. Real-world data further support meaningful responses in heavily pretreated HER2-positive gynecologic malignancies [84]. The bystander killing effect of T-DXd, enabled by its membrane-permeable payload, may help overcome intratumoral HER2 heterogeneity [85].

### ADCs targeting NaPi2b

#### Lifastuzumab vedotin (LIFA)

LIFA, an anti-NaPi2b ADC conjugated to monomethyl auristatin E (MMAE) via a cleavable mc-val-cit-PABC linker, demonstrated initial antitumor activity in OC. Early-phase studies (NCT01363947) reported an ORR of up to 46% with monotherapy and 59% with carboplatin ± bevacizumab, with a median PFS of 10.7 months [86]. However, high rates of grade ≥ 3 treatment-related adverse events (TRAEs), including pulmonary toxicity, limited its tolerability [87]. A subsequent phase II trial (NCT01991210) showed modest improvements over pegylated liposomal doxorubicin (PLD) but insufficient durability of response, leading to discontinuation of further development [88].

#### Upifitamab rilsodotin (UpRi)

UpRi, a NaPi2b-targeted ADC with a monomethyl auristatin F (MMAF) payload, showed manageable safety and preliminary efficacy in a phase I trial (NCT03319628), achieving an ORR of 34%. However, the phase II UPLIFT trial, presented at the 2024 SGO Annual Meeting, reported limited clinical benefit, with ORRs of 15.6% in the NaPi2b-positive subgroup and 13.1% overall [89], failing to meet efficacy benchmarks and leading to program termination. UpRi is currently under evaluation in the phase III UP-NEXT trial (NCT05329545) as maintenance therapy for recurrent PSOC patients responding to platinum-based therapy [90], aiming to assess PFS prolongation in a molecularly selected population.

### ADCs targeting MSLN

Mesothelin (MSLN), a glycosylphosphatidylinositol (GPI)-anchored cell surface glycoprotein, is overexpressed in approximately 70% of OC, particularly HGSOC, making it a compelling target for ADC-based therapies. Anetumab ravtansine (AR) demonstrated promising preclinical activity, including bystander killing [91]. However, clinical outcomes have been mixed. A phase Ib trial (NCT02751918) of AR combined with PLD achieved a 27.7% ORR in patients with PROC [91]. In contrast, a phase II trial comparing AR plus bevacizumab (ARB) to paclitaxel plus bevacizumab (PB) reported inferior PFS (5.3 vs. 12.7 months) and lower ORR (21% vs. 65%) in the ARB arm, leading to early termination [92]. Another MSLN-targeted ADC, DMOT4039A, showed limited efficacy in a phase I trial (NCT01469793), with partial responses observed in a small subset of patients [93].

### ADCs targeting DPEP3

DPEP3, a GPI-anchored protease, is minimally expressed in normal tissues but upregulated in OC cells, including tumor-initiating subpopulations, making it a potential ADC target [94]. Tamrintamab pamozirine (SC-003), a DPEP3-targeted ADC, was evaluated in a phase I dose-escalation and expansion trial (NCT02539719, *n* = 74) in DPEP3-positive OC. The ORR was only 4%, with non-durable responses, indicating limited antitumor activity [94].

### ADCs targeting TF

Tissue factor (TF), a transmembrane glycoprotein involved in coagulation, is overexpressed in a subset of OC, particularly high-grade tumors, making it a promising target for ADC therapy [95]. Tisotumab vedotin (TV), a TF-targeted ADC with a MMAE payload, is FDA-approved for recurrent/metastatic cervical cancer and has been evaluated in OC. The phase I/II InnovaTV 201 trial (NCT02001623) reported a modest ORR of 15.6% in heavily pretreated solid tumors, including OC, with manageable toxicities such as fatigue, nausea, and ocular adverse events [95]. The ongoing phase II InnovaTV 208 trial (NCT03657043) is specifically assessing TV in PROC, with final results pending [96]. These findings underscore the therapeutic potential of TF-targeted ADCs while highlighting the need for biomarker-driven patient selection to optimize clinical benefit.

### ADCs targeting CDH6

The phase II/III REJOICE-Ovarian01 trial (NCT06161025) is evaluating raludotatug deruxtecan (R-DXd), a CDH6-targeted ADC, in high-grade serous or endometrioid OC and related gynecologic cancers. Following promising phase I activity, the study aims to optimize dosing in phase II and compare R-DXd to investigator’s choice chemotherapy in phase III, with primary endpoints of ORR and PFS. Phase I data showed a 46% ORR and a median DoR of 11.2 months in heavily pretreated patients [97, 98].

Importantly, the integration of molecular profiling, targeting FRα, HER2, MSLN, NaPi2b, TF, CDH6, and emerging targets like DPEP3, will be critical for optimizing patient selection and treatment sequencing in ADC-based therapies. Ongoing clinical and regulatory advancements are expected to further refine personalized ADC-based therapeutic strategies for OC **(**Table 5**)**.

### Mechanisms of resistance to ADCs

ADC resistance in OC stems from multiple mechanisms: antigen modulation, including downregulation, shedding, or mutation of target antigens, diminishes binding; endocytic defects impair internalization or lysosomal processing, reducing payload release; efflux pump overexpression, such as MDR1 upregulation, lowers intracellular payload levels, as seen in tisotumab vedotin; and TME-mediated resistance, driven by hypoxia, stromal barriers, or immunosuppressive cells, hinders ADC delivery and efficacy. Understanding these resistance pathways is crucial for the development of rational combination strategies and next-generation ADCs [99]. Integration of serial tumor biopsies and molecular profiling in ongoing and future studies will be critical to elucidate predictive biomarkers and resistance pathways. Combining T-DXd with agents targeting complementary resistance mechanisms, for example, efflux inhibitors, ICIs, represents a compelling avenue for enhancing efficacy [100].

### Antigen heterogeneity and histologic subtypes

The histologic diversity of OC drives variable antigen expression, impacting ADC efficacy. FRα is highly expressed in HGSOC but less in endometrioid and mucinous subtypes, while HER2 is more prevalent in clear cell and mucinous OC. NaPi2b, MSLN, and TF expression also varies across subtypes, necessitating subtype-specific biomarker stratification [101]. The TME may also modulate ADC efficacy. High stromal content and poor vascularization can impair ADC delivery, while immune contexture may influence payload-induced immunogenic cell death.

The efficacy of ADCs in OC depends on stable target expression and overcoming histologic heterogeneity and resistance. Emerging platforms, including bispecific, bystander-enhanced, and tumor microenvironment (TME)-adaptive ADCs, alongside biomarker-guided patient selection and rational combination strategies, are critical for maximizing their clinical impact.

## Immunotherapies for the treatment of OC

Over the past two decades, immunotherapy has revolutionized cancer treatment, spurring investigation into its potential for OC. Despite limited clinical responses, ongoing research into immune checkpoint inhibitors (ICIs), CAR-T cell therapy, and tumor vaccines continues to seek effective strategies for OC.

### Immune checkpoint inhibitors (ICIs)

**Fig. 4:**
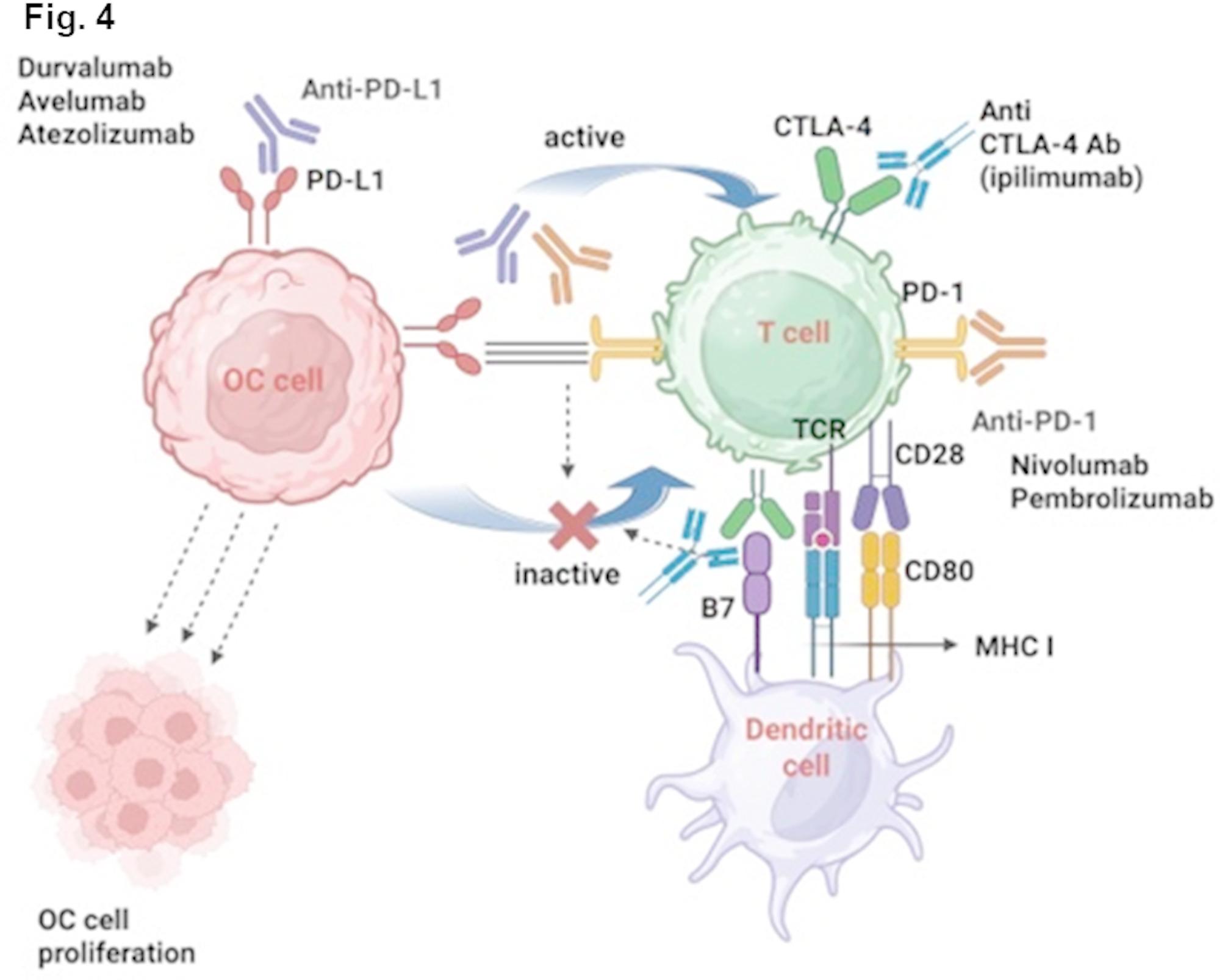
Mechanism of ICIs for OC treatment. PD-1/PD-L1 pathway: OC cells express PD-L1 on their surface, which binds to PD-1 receptors on T cells, thereby inhibiting T cell activity. Anti-PD-1 and anti-PD-L1 antibodies can block this interaction, restoring the cytotoxic activity of T cells. CTLA-4/B7 Pathway: Dendritic cells express B7 molecules on their surface, which bind to CTLA-4 on T cells, inhibiting T cell activity. Anti-CTLA-4 antibodies can block this interaction, enhancing T cell activity. MHC/TCR Pathway: Dendritic cells present antigens via MHC class I molecules, which activate T cell receptors (TCR), further activating T cells

ICIs target programmed cell death ligand 1 (PD-L1) on tumor cells, programmed cell death 1 (PD-1), and cytotoxic T lymphocyte-associated protein 4 (CTLA-4) on T cells to enhance anti-tumor immunity in OC [102]. In the PD-1/PD-L1 pathway, PD-1 interacts with its ligand PD-L1, leading to T-cell suppression and tumor immune evasion. Anti-PD-1 antibodies (e.g., Pembrolizumab) and anti-PD-L1 antibodies (e.g., Atezolizumab) block this pathway, restoring T-cell activity against tumors. In the CTLA-4 pathway, CTLA-4 functions as an inhibitory receptor, primarily suppressing T-cell activation to prevent excessive immune responses. Anti-CTLA-4 antibodies, such as Ipilimumab, block CTLA-4, thereby enhancing T-cell activation and strengthening antitumor immune responses [103] (Fig. 4). ICIs have limited efficacy as monotherapies in unselected OC populations, typically achieving ORR below 15%. However, ICIs have shown promising outcomes in biomarker-enriched subgroups and in combination regimens [104]. Notably, dual blockade (e.g., nivolumab plus ipilimumab) and triplet therapies incorporating anti-angiogenic or chemotherapeutic agents have demonstrated enhanced clinical benefit.

#### Immune landscape in OC

The immune microenvironment in OC is highly heterogeneous, impacting prognosis and immunotherapy response. Elevated intraepithelial CD8⁺ tumor-infiltrating lymphocytes (TILs) are linked to improved survival [105], while increased regulatory T cells (Tregs) and M2 macrophages are associated with immune suppression and poorer outcomes. Tumors often upregulate PD-L1 in response to local IFN-γ, promoting immune evasion [106]. Most OC tumors exhibit an immune “cold” or “excluded” phenotype, characterized by limited T-cell infiltration into the tumor epithelium and a relatively low tumor mutational burden (TMB, median 3.6 mutations/Mb) [107], substantially lower than in melanoma or lung cancer. These immunologic features contribute to the limited efficacy of ICI monotherapy in OC. However, the presence of TILs in select tumors and biomarkers such as microsatellite instability-high (MSI-H) or HRD suggest that rationally designed combination strategies may still elicit robust immune responses.

#### ICIs trials in OC

##### Monotherapy trials

ICI monotherapy has shown limited efficacy in unselected OC populations. Early studies of avelumab, nivolumab, and pembrolizumab reported ORR of 9–10% in recurrent OC or platinum-resistant OC (PROC) [108]. The phase II KEYNOTE-100 trial demonstrated an ORR of 8.5% with pembrolizumab, increasing to 14.1% in patients with PD-L1 combined positive score (CPS) ≥ 10 [109]. Similarly, ipilimumab monotherapy achieved an ORR of 10.3% in platinum-sensitive OC (PSOC) post-chemotherapy [110, 111]. These findings highlight the need for biomarker-driven patient selection. Notably, pembrolizumab is FDA-approved for MSI-H or mismatch repair-deficient (dMMR) OC, regardless of tumor origin, due to durable responses in this immunogenic subgroup.

##### Combination strategies

Dual checkpoint blockade with nivolumab and ipilimumab in the NRG-GY003 trial significantly improved ORR (31.4% vs. 12.2%) and PFS (3.9 vs. 2.0 months) compared to nivolumab alone, though with increased toxicity [112]. ICI plus anti-angiogenic therapy has shown promise. A phase II trial combining pembrolizumab, bevacizumab, and metronomic cyclophosphamide in PROC achieved an ORR of 47.5%, a disease control rate (DCR) of 95%, and a mean PFS of 10.0 months [113]. ICI plus PARP inhibitor combinations are also compelling. In the MEDIOLA and TOPACIO trials, durvalumab or pembrolizumab combined with olaparib or niraparib yielded ORR of 14–18%, with activity in both BRCA-mutant and wild-type tumors [114], likely due to enhanced neoantigen exposure and immune priming from PARP inhibition [115]. In contrast, ICI-chemotherapy combinations have underperformed. The phase III IMagyn050 and JAVELIN-200 trials, evaluating atezolizumab and avelumab, respectively, failed to show PFS or OS benefits in newly diagnosed OC or PROC [116], despite exploratory signals of benefit in PD-L1-positive subsets [117].

Overall, current evidence supports rational ICIs combinations with TME-modulating agents, such as anti-angiogenic drugs and PARP inhibitors, particularly in biomarker-defined subgroups. Pembrolizumab for MSI-H/dMMR OC remains the only FDA-approved ICI-based therapy, but ongoing trials may soon expand indications.

#### Predictive biomarkers of ICIs response in OC

Key biomarkers for ICIs response in OC include PD-L1 expression, tumor mutational burden (TMB), homologous recombination deficiency (HRD), and gene expression signatures.

**PD-L1 expression:** PD-L1 is the most widely studied biomarker, but its predictive value in OC remains inconsistent. The KEYNOTE-100 trial reported higher ORR with pembrolizumab in patients with PD-L1 combined positive score (CPS) ≥ 10 (14.1% vs. 4.1%) [118]. Recent spatial profiling studies further highlight that PD-L1 distribution within immune-infiltrated tumor regions is more informative than overall expression levels, as “diffuse infiltration” areas with elevated PD-L1, PD-1, and TIM-3 are associated with better ICI responses [119, 120].

**TMB:** TMB is an established pan-cancer predictor of ICIs benefit, but most OC tumors are TMB-low (median 3.6 mutations/Mb) [121–123]. In the IMagyn050 biomarker cohort, only 3% of tumors met the TMB-high threshold (≥ 10 mutations/Mb), and 0.3% were MSI-H [124]. Accordingly, the FDA has approved pembrolizumab for MSI-H/MMRd OC regardless of histology, but TMB is not a routinely used biomarker in this setting.

**HRD:** Present in approximately 50% of HGSOC, HRD is linked to higher neoantigen load and immune checkpoint expression [125, 126], yet often lacks sufficient T-cell infiltration or immune activation [127]. Trials such as TOPACIO (niraparib + pembrolizumab) and the olaparib-durvalumab study reported responses irrespective of BRCA/HRD status [128], suggesting HRD alone does not predict ICIs sensitivity but may enhance efficacy in combination with PARP inhibitors through increased neoantigen exposure.

**Other biomarkers:** Emerging biomarkers include high CD8⁺ TIL density [129], IFN-γ-related gene expression profiles, and the immunoreactive molecular subtype identified by The Cancer Genome Atlas (TCGA) [130, 131], which exhibits elevated immune gene expression and may predict ICIs responsiveness independent of HRD. POLE mutations, though rare in OC, may increase TMB and immunogenicity [132]. Currently, only MSI-H, dMMR, and PD-L1 CPS ≥ 10 serve as actionable biomarkers in clinical practice [133].

#### Mechanisms of immune evasion and ICIs resistance in OC

OC employs diverse immune evasion strategies that reduce the efficacy of ICIs. These include upregulation of inhibitory ligands, recruitment of immunosuppressive cells, impaired antigen presentation, and checkpoint-independent resistance mechanisms.

**PD-L1 upregulation and cytotoxic T lymphocyte (CTL) exhaustion**.

PD-L1 upregulation on OC tumor and stromal cells, often induced by IFN-γ from infiltrating lymphocytes during peritoneal dissemination, is a primary immune evasion mechanism [134]. Abiko et al. showed that lymphocyte interactions drive PD-L1 expression, suppressing CTL activation and inducing an exhausted CTL profile [135]. This PD-L1 overexpression reduces CTL degranulation, promoting immune escape [106]. Murine models demonstrated that PD-L1 ablation attenuates peritoneal metastasis and extends survival, highlighting its role [136]. This tumor-immune feedback loop, where initial immune responses enhance PD-L1-mediated suppression, supports the use of anti-PD-1/PD-L1 antibodies to restore CTL function, a key rationale for early ICI trials in OC.

**Immunosuppressive cytokines and cells**.

The OC TME is highly immunosuppressive, driven by cytokines, metabolic enzymes, and immune cell populations. TGF-β1, abundant in malignant ascites, promotes Treg differentiation, impairs natural killer (NK) cell cytotoxicity, and inhibits effector T cell activation [137]. IL-10 from tumor-associated macrophages suppresses antigen presentation and T cell priming [138]. Overexpressed indoleamine 2,3-dioxygenase (IDO) depletes tryptophan, inducing T cell anergy. MDSCs and immunosuppressive neutrophils, recruited via vascular endothelial growth factor (VEGF)/ vascular endothelial growth factor receptor 2 (VEGFR2) signaling, are prevalent in ascitic fluid [139]. Tregs, attracted by CCL22, correlate with poor prognosis [140]. Adenosine, generated by CD39 and CD73, further inhibits T cell function [141]. These factors create a robust immunosuppressive TME, limiting anti-tumor immunity.

**Tumor heterogeneity and antigenicity**.

Many OCs, particularly HGSCs, exhibit low neoantigen loads, limiting immunogenicity. Except in rare MSI-H or POLE-mutant cases, HGSCs generate few immunogenic peptides [142]. Even in HRD tumors, which have higher neoantigen potential, impaired antigen presentation, via HLA class I downregulation or β2-microglobulin mutations, prevents robust immune responses [142]. Intratumoral heterogeneity further promotes immune-resistant subclones under selective pressure, contributing to “immune desert” or “immune-excluded” TME [143], characterized by absent T-cell infiltration or T cells confined to stromal compartments [144]. These features hinder ICI efficacy in OC.

**Checkpoint-independent resistance**.

Primary ICI resistance in OC often involves alternative inhibitory receptors (e.g., LAG-3, TIM-3, TIGIT), which sustain T cell exhaustion beyond PD-1/PD-L1 blockade [145, 146]. Oncogenic pathways like PI3K/AKT or Wnt/β-catenin signaling suppress T cell infiltration and antigen presentation, with β-catenin-driven dendritic cell exclusion (observed in melanoma) being potentially active in OC [145, 146]. Hypoxia in the peritoneal TME upregulates VEGF, and IL-10 and downregulates MHC class I via HIF-1α, reducing immune recognition. These mechanisms necessitate combination therapies targeting both immune and tumor-intrinsic pathways in order to overcome ICI resistance in OC.

#### Strategies to modulate the TME for improved ICI efficacy

Combining ICIs with chemotherapy, PARP inhibitors (PARPis), and anti-angiogenic agents aims to reprogram the immunosuppressive TME and enhance ICI efficacy in OC.

**Chemotherapy and ICIs**.

Chemotherapy can induce immunogenic cell death, releasing neoantigens and depleting immunosuppressive cells to enhance dendritic cell activation. Anthracyclines and taxanes are particularly effective, but high-dose regimens may deplete lymphocytes, reducing efficacy. The phase III IMagyn050 trial, which combined atezolizumab with carboplatin-paclitaxel-bevacizumab in frontline OC, showed no PFS or OS improvement, indicating that simple ICI-chemotherapy combinations are insufficient [147]. Sequencing strategies, such as administering pembrolizumab post-neoadjuvant chemotherapy to leverage treatment-induced inflammation, are under exploration. Ongoing trials, such as ENGOT-ov65/KEYNOTE-B96 (pembrolizumab + paclitaxel ± bevacizumab), aim to clarify the role of ICIs in lower-toxicity salvage regimens [148].

**PARP inhibitors**.

PARP inhibition upregulates PD-L1 expression and activates the cGAS-STING pathway, promoting type I interferon signaling and an inflamed TME, sensitizing tumors to ICIs. The combination of olaparib and durvalumab demonstrated immunologic modulation and modest antitumor activity in OC [149]. Similarly, niraparib plus pembrolizumab yielded responses in some non-HRD tumors, with a median DoR not reached in one trial, suggesting durable benefits [150]. As PARPi maintenance therapy is standard for BRCA-mutated or HRD-positive OC, combining PARPis with PD-1/PD-L1 inhibitors offers a strategy to enhance responses. Phase III trials, including MEDIOLA and FIRST, are evaluating this approach [151]. Preliminary FIRST trial results showed modest PFS improvements with dostarlimab, chemotherapy, and niraparib compared to chemotherapy plus niraparib alone, with greater benefits in PD-L1-positive tumors [152]. These data highlight the synergy between DNA repair inhibition and immunotherapy.

**Anti-angiogenic agents**.

VEGF drives angiogenesis and immunosuppression via MDSCs recruitment and Treg expansion. Bevacizumab enhances ICI efficacy by normalizing tumor vasculature, increasing T-cell infiltration, and reducing MDSCs. The phase I PEMBOV study of pembrolizumab plus bevacizumab in PROC reported a 26% ORR and 79% DCR without chemotherapy [113]. High VEGFR3 levels in the olaparib + durvalumab trial correlated with poorer PFS, suggesting angiogenic signaling hinders immune responses [153]. Upcoming trials are integrating bevacizumab with PARPi-ICI regimens [153]. Multitargeted inhibitors like lenvatinib (targeting VEGFR/FGFR) combined with pembrolizumab showed a 35% ORR and 6.2-month median PFS in heavily pretreated OC in the LEAP-005 study, independent of PD-L1 status [153]. A case series in ovarian clear cell carcinoma reported 100% response with durable benefits [154]. These results support combining VEGF inhibitors with ICIs, and potentially PARPis, to reprogram the TME and enhance anti-tumor immunity in OC.

#### CTLA-4 blockade and other checkpoints

Dual checkpoint blockade with nivolumab and ipilimumab has shown enhanced efficacy in OC. The randomized phase II NRG-GY003 trial reported a doubled ORR for the combination (31.4%) compared to nivolumab alone (12.2%), with prolonged PFS, though with higher toxicity [112]. In clear-cell OC, the phase II BrUOG 354 study demonstrated a 33.3% ORR and 24.7-month median OS with nivolumab plus ipilimumab, versus 14.3% and 17.3 months for monotherapy. Alternative checkpoints, such as LAG-3 and TIM-3, are under investigation to address T-cell exhaustion. While a melanoma trial combining PD-1 and IDO inhibitors was negative, IDO1 modulation is being explored in OC due to its role in T-cell suppression via tryptophan catabolism. Bispecific or multispecific antibodies targeting multiple inhibitory pathways simultaneously are in early development, offering a promising approach to enhance ICI efficacy in OC.

In conclusion, despite limited efficacy of ICIs as monotherapy in OC (ORR < 15%), select subtypes, particularly clear cell and endometrioid tumors with MSI-H or ARID1A mutations, exhibit greater immunogenicity and responsiveness. Biomarker-guided combination strategies, such as dual checkpoint blockade or ICIs with PARP inhibitors and anti-angiogenic agents, have demonstrated improved outcomes by modulating the immunosuppressive TME and converting “cold” tumors into immunologically active ones.

### Chimeric antigen receptor T (CAR-T) cell therapy

**Fig. 5:**
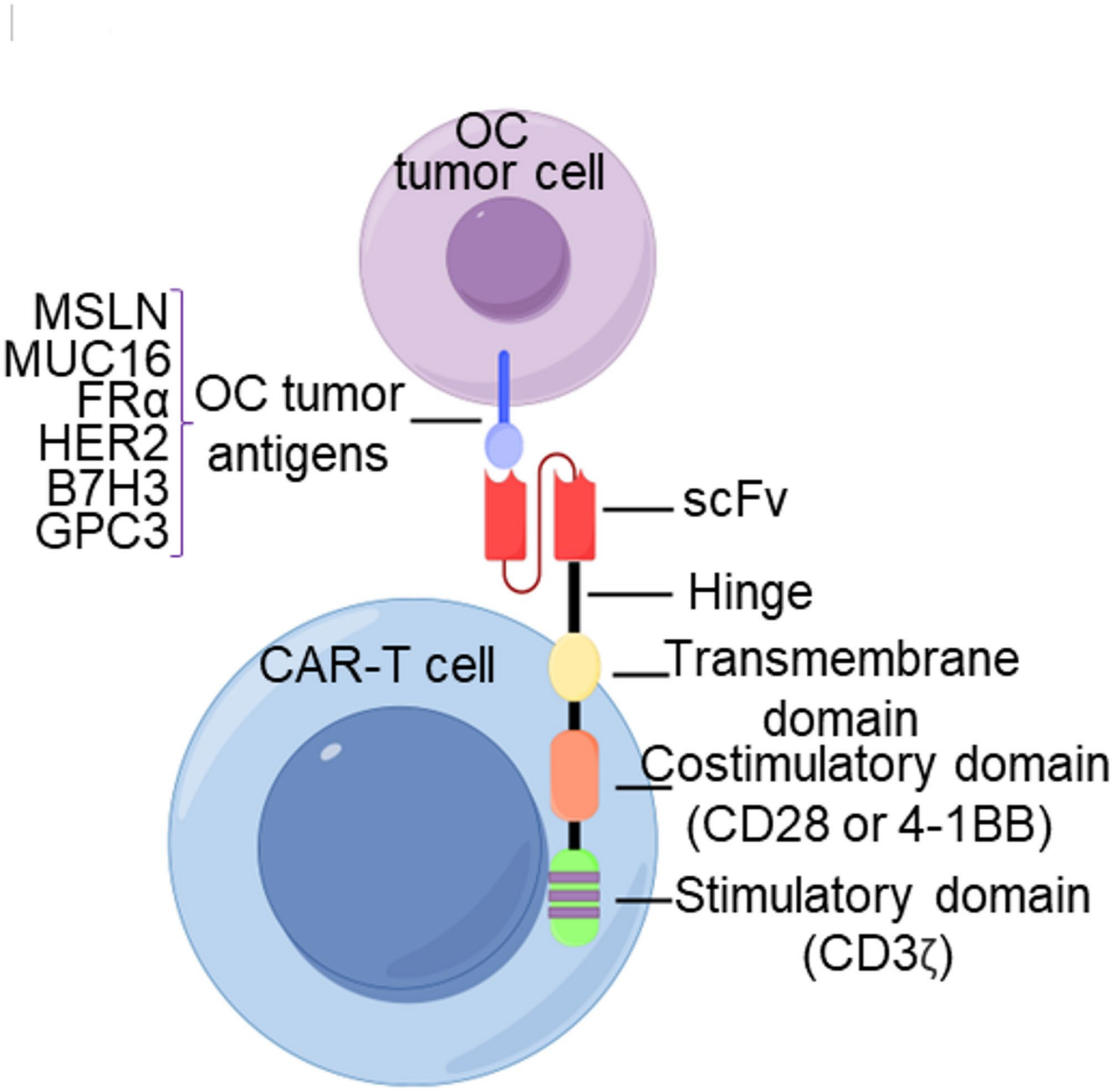
Mechanism of CAR-T cell therapy for OC treatment. Synthetic chimeric antigen receptor T (CAR-T) cells recognize the tumor-associated antigens, including mesothelin (MSLN), MUC16, folate receptor alpha (FRα), human epidermal growth factor receptor 2 (HER2), B7H3 and glypican-3 (GPC3), on the surface of OC tumor cells via their single-chain variable fragment (scFv) domain. This interaction triggers both CD3-mediated primary stimulatory signaling and CD28/4-1BB-mediated secondary co-stimulatory signaling, leading to CAR-T cell activation and anti-tumor response

CAR-T cell therapy is an innovative immunotherapy that involves genetically modifying T cells to enable them to recognize and eliminate tumor cells (Fig. 5). While highly effective in hematologic malignancies, its application in solid tumors like OC faces challenges due to tumor heterogeneity, the immunosuppressive TME, limited T-cell infiltration, and toxicity. Advances in regional delivery, combination therapies, engineered CAR constructs, and biomarker-driven patient selection are critical for improving efficacy in OC.

#### Target antigens and structural innovations

CAR-T therapies for OC target tumor-associated antigens, including mesothelin (MSLN), MUC16, folate receptor alpha (FRα), human epidermal growth factor receptor 2 (HER2), B7H3, and glypican-3 (GPC3) (Fig. 5). MSLN is overexpressed in 70% of AOC [155]. Novel multichain DAP-CAR constructs, incorporating NK-ligand interactions, enhance in vitro cytotoxicity and in vivo efficacy in xenograft models compared to conventional single-chain variable fragment (scFv)-based CARs [156]. Humanized scFv designs targeting membrane-proximal MSLN epitopes (e.g., region III binders like G11 hYP218) improve tumor killing and minimize shedding interference [157].

#### Clinical efficacy

In the phase I ChiCTR2100046544 study, eight OC patients received intravenous MSLN-DAP-CAR-T, achieving PR in two patients and stable disease (SD) in four, with a 100% DCR at 3 months [158]. Early MSLN-BBZ CAR-T trials showed T-cell expansion but limited antitumor activity [159]. Overall, CAR-T therapies in OC yield promising DCRs (up to 100%) but modest ORRs (25–33%). Ongoing trials target MUC16ecto, FRα, and fourth-generation CARs secreting PD-1 nanobodies (e.g., NCT05057715, NCT02498912) [160].

#### TME and delivery barriers

The OC TME, characterized by dense stroma, solid tumor architecture, and immunosuppressive mediators (e.g., TGF-β, adenosine), impairs CAR-T trafficking and induces T-cell exhaustion. Intraperitoneal delivery is being explored to enhance T-cell infiltration [160]. Preclinical advances include gene-edited CAR-T cells targeting immunosuppressive pathways (e.g., A2aR, TGF-βR) and “TRUCKs” secreting IL-7/CCL19 to boost T-cell function. Strategies like loco-regional delivery, antigen-specific targeting, extracellular matrix degradation via oncolytic viruses, and combinations with checkpoint inhibitors or anti-angiogenic agents show promise for overcoming TME barriers [160].

#### Safety and toxicity

CAR-T therapy in OC carries risks, including cytokine release syndrome (CRS) and immune effector cell-associated neurotoxicity syndrome (ICANS) [158]. On-target/off-tumor toxicity, such as pulmonary injury or sepsis, arises from antigen expression on normal mesothelium. Safety strategies under evaluation include dose titration, suicide switches, split CAR designs, and localized delivery to minimize systemic toxicity.

### Tumor vaccines

Tumor vaccines are an emerging immunotherapy strategy for OC, designed to deliver tumor-associated antigens into the body, thereby stimulating a specific antitumor immune response. By activating T cells and other immune components, tumor vaccines enhance the recognition and elimination of cancer cells, potentially addressing limitations of conventional therapies that often fail to eradicate tumors completely [161]. Despite growing research interest and increasing clinical trials, challenges remain, including small sample sizes and failure to meet clinical endpoints in many studies. Advances in vaccine design, antigen selection, and delivery methods offer promise for improving efficacy.

Dendritic cell (DC)-based vaccines have shown meaningful survival benefits and higher disease stabilization rates in OC [161]. To enhance tumor vaccine efficacy in OC, several strategies are under exploration. Intraperitoneal infusion is being investigated to improve antigen presentation and T-cell infiltration within the TME, overcoming barriers like dense stroma and immunosuppressive mediators [161]. Incorporating safety features, such as suicide switches, split-CAR designs, or regulated expression systems, allows higher dosing with manageable toxicity. Identifying predictive biomarkers, including MSLN, FRα, immune profiles, and vascular signatures (e.g., bevacizumab-associated transcriptomes), supports patient selection and treatment optimization [159].

### Other emerging immunotherapies and novel therapeutic for OC

Other emerging immunotherapies for OC, including oncolytic viruses and cytokine-based therapies, aim to overcome the immunosuppressive TME and enhance anti-tumor immunity. Additionally, avutometinib plus defactinib have been approved for novel therapeutic in KRAS‑mutated recurrent low‑grade serous OC.

#### Oncolytic viruses

Olvimulogene nanivacirepvec (Olvi-Vec), an oncolytic virus, is being evaluated in a phase III trial (NCT06890338) for PROC or refractory OC. Administered intraperitoneally with platinum-doublet chemotherapy and bevacizumab, Olvi-Vec aims to modulate the TME and resensitize tumors to platinum agents. Eligible patients with recurrent, non-resectable high-grade serous, endometrioid, or clear-cell OC, fallopian tube, or primary peritoneal cancer, post ≥ 3 chemotherapy lines, are randomized (2:1) to Olvi-Vec plus chemotherapy and bevacizumab or chemotherapy and bevacizumab alone. The primary endpoint is PFS, targeting 186 patients for 127 PFS events, with results expected in 2025 [162]. The dual-cytokine-armed adenovirus TILT-123, combined with pembrolizumab, showed promising disease control rates (64–71%) and immune activation in a phase I trial for PROC (NCT05271318) [163, 164].

#### Cytokine-based therapies

Nemvaleukin alfa (ALKS 4230), an engineered IL-2 cytokine, selectively activates CD8⁺ T cells and natural killer cells via intermediate-affinity IL-2 receptors, minimizing Treg expansion. In the phase I/II ARTISTRY-1 trial (NCT02839707), nemvaleukin monotherapy (6 µg/kg/day) or with pembrolizumab achieved ORRs of 10% and 13%, respectively, in 243 heavily pretreated patients with advanced solid tumors, including three responses in PROC [165]. Ongoing phase II/III trials are evaluating its efficacy in select tumor types.

#### Novel therapeutic: avutometinib plus defactinib in kras‑mutated recurrent low‑grade serous OC

In May 2025, the FDA granted accelerated approval to avutometinib plus defactinib for adult patients with KRAS‑mutated recurrent low‑grade serous ovarian cancer (LGSOC) after at least one prior systemic therapy, marking the first-ever FDA‑approved treatment for LGSOC [166]. This approval was based on the phase 2 RAMP‑201 trial (NCT04625270), which enrolled 57 heavily pretreated patients. In those with measurable disease and KRAS mutations, the confirmed ORR was 44% (95% CI: 31–58), with a median PFS of approximately 22 months and a DoR extending up to 31 months [167]. The combination of avutometinib (a RAF/MEK inhibitor) and defactinib (a focal adhesion kinase [FAK] inhibitor) targets interconnected signaling pathways driving tumorigenesis in KRAS-mutant LGSOC. The safety profile includes manageable toxicities, such as rash, elevated creatine phosphokinase, and ocular events, with recommendations for proactive dermatologic prophylaxis and regular ophthalmologic monitoring during therapy [168]. Although KRAS mutation testing is not yet included in ASCO or SGO clinical practice guidelines, this FDA approval signals a potential shift toward routine KRAS testing for LGSOC, which may prompt guideline updates to incorporate this biomarker into diagnostic workflows.

In summary, immunotherapies show promise in the treatment of OC but face multiple challenges, including low ORRs (< 15%), tumor heterogeneity, and resistance mechanisms. The immunosuppressive TME, driven by Tregs and MDSCs, further hinders the effectiveness of immunotherapy, necessitating the development of novel therapeutic strategies to overcome these barriers. Future research should prioritize the identification of novel immune checkpoint targets, the refinement of combination therapy strategies, and the development of innovative approaches to overcome immune resistance, all with the goal of enhancing treatment efficacy and improving survival outcomes for OC patients. Additionally, advancements in nanotechnology and 3D organoid models present promising opportunities to optimize immunotherapy, potentially reducing adverse effects and enhancing therapeutic responses through more precise and personalized treatment strategies.

## Conclusions and future perspectives

Despite advances in surgery, chemotherapy, targeted therapies, and immunotherapy, OC continues to face challenges of recurrence and resistance. Biomarker-guided strategies leveraging BRCA mutations, HRD status, and immune profiling, have enhanced the precision of PARP inhibitors, ADCs, ICIs, and other novel immunotherapies.

Future efforts should prioritize optimizing patient selection and developing rational combination therapies to overcome resistance. Integrating precision oncology with immunomodulatory approaches and novel agents offers promise for improving survival and transforming OC into a more manageable disease.

## Data Availability

No datasets were generated or analysed during the current study.

## References

[1] Bray F, et al. Global cancer statistics 2022: GLOBOCAN estimates of incidence and mortality worldwide for 36 cancers in 185 countries. CA Cancer J Clin. 2024;74(3):229–63. 38572751 10.3322/caac.21834

[2] Siegel RL, Miller KD, Jemal A. Cancer statistics, 2019. CA Cancer J Clin. 2019;69(1):7–34. 30620402 10.3322/caac.21551

[3] Vergote I, et al. Neoadjuvant chemotherapy or primary surgery in stage IIIC or IV ovarian cancer. N Engl J Med. 2010;363(10):943–53. 20818904 10.1056/NEJMoa0908806

[4] du Bois A, et al. Role of surgical outcome as prognostic factor in advanced epithelial ovarian cancer: a combined exploratory analysis of 3 prospectively randomized phase 3 multicenter trials: by the arbeitsgemeinschaft gynaekologische onkologie studiengruppe ovarialkarzinom (AGO-OVAR) and the groupe d’investigateurs Nationaux pour les etudes des cancers de l’ovaire (GINECO). Cancer. 2009;115(6):1234–44. 19189349 10.1002/cncr.24149

[5] Fagotti A, et al. Prospective validation of a laparoscopic predictive model for optimal cytoreduction in advanced ovarian carcinoma. Am J Obstet Gynecol. 2008;199(6):e6421–6. 10.1016/j.ajog.2008.06.052 18801470

[6] Suidan RS, et al. A multicenter assessment of the ability of preoperative computed tomography scan and CA-125 to predict gross residual disease at primary debulking for advanced epithelial ovarian cancer. Gynecol Oncol. 2017;145(1):27–31. 28209497 10.1016/j.ygyno.2017.02.020 PMC5387995

[7] Aletti GD, et al. Identification of patient groups at highest risk from traditional approach to ovarian cancer treatment. Gynecol Oncol. 2011;120(1):23–8. 20933255 10.1016/j.ygyno.2010.09.010

[8] Vizzielli G, et al. A laparoscopic risk-adjusted model to predict major complications after primary debulking surgery in ovarian cancer: A single-institution assessment. Gynecol Oncol. 2016;142(1):19–24. 27103179 10.1016/j.ygyno.2016.04.020

[9] Jonsdottir B, et al. The peritoneal cancer index is a strong predictor of incomplete cytoreductive surgery in ovarian cancer. Ann Surg Oncol. 2021;28(1):244–51. 32472412 10.1245/s10434-020-08649-6 PMC7752870

[10] Eisenkop SM, et al. Relative influences of tumor volume before surgery and the cytoreductive outcome on survival for patients with advanced ovarian cancer: a prospective study. Gynecol Oncol. 2003;90(2):390–6. 12893206 10.1016/s0090-8258(03)00278-6

[11] Dessapt AL, et al. Is complete cytoreductive surgery feasible in this patient with ovarian cancer? Surg Oncol. 2016;25(3):326–31. 27566040 10.1016/j.suronc.2016.07.001

[12] Pignata S, et al. Carboplatin-based doublet plus bevacizumab beyond progression versus carboplatin-based doublet alone in patients with platinum-sensitive ovarian cancer: a randomised, phase 3 trial. Lancet Oncol. 2021;22(2):267–76. 33539744 10.1016/S1470-2045(20)30637-9

[13] Pfisterer J, et al. Bevacizumab and platinum-based combinations for recurrent ovarian cancer: a randomised, open-label, phase 3 trial. Lancet Oncol. 2020;21(5):699–709. 32305099 10.1016/S1470-2045(20)30142-X

[14] Taguchi A, et al. Heterogeneous treatment effect of dose-dense Paclitaxel plus carboplatin therapy for advanced ovarian cancer. Int J Cancer. 2024;155(6):1068–77. 38712630 10.1002/ijc.34996

[15] Pignata S, et al. Carboplatin plus Paclitaxel once a week versus every 3 weeks in patients with advanced ovarian cancer (MITO-7): a randomised, multicentre, open-label, phase 3 trial. Lancet Oncol. 2014;15(4):396–405. 24582486 10.1016/S1470-2045(14)70049-X

[16] Clamp AR, et al. Weekly dose-dense chemotherapy in first-line epithelial ovarian, fallopian tube, or primary peritoneal cancer treatment (ICON8): overall survival results from an open-label, randomised, controlled, phase 3 trial. Lancet Oncol. 2022;23(7):919–30. 35690073 10.1016/S1470-2045(22)00283-2 PMC9630160

[17] Banerjee S, et al. A multicenter, randomized trial of flat dosing versus intrapatient dose escalation of single-agent carboplatin as first-line chemotherapy for advanced ovarian cancer: an SGCTG (SCOTROC 4) and ANZGOG study on behalf of GCIG. Ann Oncol. 2013;24(3):679–87. 23041585 10.1093/annonc/mds494 PMC4669851

[18] Fagotti A, et al. Minimally invasive secondary cytoreduction plus HIPEC versus open surgery plus HIPEC in isolated relapse from ovarian cancer: a retrospective cohort study on perioperative outcomes. J Minim Invasive Gynecol. 2015;22(3):428–32. 25461683 10.1016/j.jmig.2014.11.008

[19] May T. Secondary cytoreductive surgery with HIPEC: a promising therapeutic option for recurrent ovarian cancer. Lancet Oncol. 2024;25(12):1509–11. 39549721 10.1016/S1470-2045(24)00592-8

[20] Steadman JA, Grotz TE. Principles of surgical management of peritoneal mesothelioma. J Natl Compr Canc Netw. 2023;21(9):981–6. 37673112 10.6004/jnccn.2023.7055

[21] Filis P, et al. Hyperthermic intraperitoneal chemotherapy (HIPEC) for the management of primary advanced and recurrent ovarian cancer: a systematic review and meta-analysis of randomized trials. ESMO Open. 2022;7(5):100586. 36116421 10.1016/j.esmoop.2022.100586 PMC9588894

[22] Lim MC, et al. Survival after hyperthermic intraperitoneal chemotherapy and primary or interval cytoreductive surgery in ovarian cancer: A randomized clinical trial. JAMA Surg. 2022;157(5):374–83. 35262624 10.1001/jamasurg.2022.0143 PMC8908225

[23] van Driel WJ, Koole SN, Sonke GS. Hyperthermic intraperitoneal chemotherapy in ovarian cancer. N Engl J Med. 2018;378(14):1363–4. 29617590 10.1056/NEJMc1802033

[24] Aronson SL, et al. Cytoreductive surgery with or without hyperthermic intraperitoneal chemotherapy in patients with advanced ovarian cancer (OVHIPEC-1): final survival analysis of a randomised, controlled, phase 3 trial. Lancet Oncol. 2023;24(10):1109–18. 37708912 10.1016/S1470-2045(23)00396-0

[25] Fagotti A, et al. Cytoreductive surgery plus HIPEC in platinum-sensitive recurrent ovarian cancer patients: a case-control study on survival in patients with two year follow-up. Gynecol Oncol. 2012;127(3):502–5. 23022234 10.1016/j.ygyno.2012.09.020

[26] Safra T, et al. Cytoreduction surgery with hyperthermic intraperitoneal chemotherapy in recurrent ovarian cancer improves progression-free survival, especially in BRCA-positive patients- a case-control study. J Surg Oncol. 2014;110(6):661–5. 24962381 10.1002/jso.23688

[27] Bakrin N, et al. Cytoreductive surgery and hyperthermic intraperitoneal chemotherapy (HIPEC) for persistent and recurrent advanced ovarian carcinoma: a multicenter, prospective study of 246 patients. Ann Surg Oncol. 2012;19(13):4052–8. 22825772 10.1245/s10434-012-2510-4

[28] Le Brun JF, et al. Survival benefit of hyperthermic intraperitoneal chemotherapy for recurrent ovarian cancer: a multi-institutional case control study. Ann Surg Oncol. 2014;21(11):3621–7. 24819120 10.1245/s10434-014-3693-7

[29] Ehmann S, Zivanovic O, Chi DS. Why was GOG-0213 a negative trial? J Gynecol Oncol. 2021;32(1):e19. 33300314 10.3802/jgo.2021.32.e19 PMC7767651

[30] Classe JM, et al. Hyperthermic intraperitoneal chemotherapy for recurrent ovarian cancer (CHIPOR): a randomised, open-label, phase 3 trial. Lancet Oncol. 2024;25(12):1551–62. 39549720 10.1016/S1470-2045(24)00531-X

[31] Fagotti A et al. Hyperthermic intraperitoneal chemotherapy in Platinum-Sensitive recurrent ovarian cancer: A randomized trial on survival evaluation (HORSE; MITO-18). J Clin Oncol, 2025: 43(7):852–860. 10.1200/JCO.24.00686 39571127

[32] Pavone M, et al. Efficacy and safety of pressurized intraperitoneal aerosol chemotherapy (PIPAC) in ovarian cancer: a systematic review of current evidence. Arch Gynecol Obstet. 2024;310(4):1845–56. 38879697 10.1007/s00404-024-07586-z PMC11392984

[33] Alyami M, et al. Pressurised intraperitoneal aerosol chemotherapy: rationale, evidence, and potential indications. Lancet Oncol. 2019;20(7):e368–77. 31267971 10.1016/S1470-2045(19)30318-3

[34] Li N, et al. An Open-label, multicenter, Single-arm, phase II study of fluzoparib in patients with germline BRCA1/2 mutation and Platinum-sensitive recurrent ovarian cancer. Clin Cancer Res. 2021;27(9):2452–8. 33558426 10.1158/1078-0432.CCR-20-3546

[35] Mirza MR, et al. Niraparib maintenance therapy in Platinum-Sensitive, recurrent ovarian cancer. N Engl J Med. 2016;375(22):2154–64. 27717299 10.1056/NEJMoa1611310

[36] Poveda A, et al. Olaparib tablets as maintenance therapy in patients with platinum-sensitive relapsed ovarian cancer and a BRCA1/2 mutation (SOLO2/ENGOT-Ov21): a final analysis of a double-blind, randomised, placebo-controlled, phase 3 trial. Lancet Oncol. 2021;22(5):620–31. 33743851 10.1016/S1470-2045(21)00073-5

[37] Hettle R, et al. Generating health state utility values from Fact-Ovarian data collected in a phase Ii maintenance study in platinum sensitive recurrent ovarian cancer (Study 19): A comparison of mapping algorithms. Value Health. 2014;17(7):A646. 27202324 10.1016/j.jval.2014.08.2341

[38] Moore K, et al. Maintenance Olaparib in patients with newly diagnosed advanced ovarian cancer. N Engl J Med. 2018;379(26):2495–505. 30345884 10.1056/NEJMoa1810858

[39] Banerjee S, et al. Maintenance Olaparib for patients with newly diagnosed advanced ovarian cancer and a BRCA mutation (SOLO1/GOG 3004): 5-year follow-up of a randomised, double-blind, placebo-controlled, phase 3 trial. Lancet Oncol. 2021;22(12):1721–31. 34715071 10.1016/S1470-2045(21)00531-3

[40] DiSilvestro P, et al. Overall survival with maintenance Olaparib at a 7-Year Follow-Up in patients with newly diagnosed advanced ovarian cancer and a BRCA mutation: the SOLO1/GOG 3004 trial. J Clin Oncol. 2023;41(3):609–17. 36082969 10.1200/JCO.22.01549 PMC9870219

[41] Ray-Coquard I, et al. Olaparib plus bevacizumab as First-Line maintenance in ovarian cancer. N Engl J Med. 2019;381(25):2416–28. 31851799 10.1056/NEJMoa1911361

[42] Ray-Coquard I, et al. Olaparib plus bevacizumab first-line maintenance in ovarian cancer: final overall survival results from the PAOLA-1/ENGOT-ov25 trial. Ann Oncol. 2023;34(8):681–92. 37211045 10.1016/j.annonc.2023.05.005

[43] Penson RT, et al. Olaparib versus nonplatinum chemotherapy in patients with Platinum-Sensitive relapsed ovarian cancer and a germline BRCA1/2 mutation (SOLO3): A randomized phase III trial. J Clin Oncol. 2020;38(11):1164–74. 32073956 10.1200/JCO.19.02745 PMC7145583

[44] Trillsch F, et al. Efficacy and safety of Olaparib according to age in BRCA1/2-mutated patients with recurrent platinum-sensitive ovarian cancer: analysis of the phase III SOLO2/ENGOT-Ov21 study. Gynecol Oncol. 2022;165(1):40–8. 35115180 10.1016/j.ygyno.2022.01.024

[45] Tuninetti V, et al. Long-term outcomes of PARP inhibitors in ovarian cancer: survival, adverse events, and post-progression insights. ESMO Open. 2024;9(11):103984. 39541620 10.1016/j.esmoop.2024.103984 PMC11613435

[46] Freyer G, et al. Author correction: bevacizumab, olaparib, and durvalumab in patients with relapsed ovarian cancer: a phase II clinical trial from the GINECO group. Nat Commun. 2024;15(1):4753. 38834579 10.1038/s41467-024-48915-9 PMC11150369

[47] Kim YN, et al. Triplet maintenance therapy of olaparib, pembrolizumab and bevacizumab in women with BRCA wild-type, platinum-sensitive recurrent ovarian cancer: the multicenter, single-arm phase II study OPEB-01/APGOT-OV4. Nat Commun. 2023;14(1):5476. 37673858 10.1038/s41467-023-40829-2 PMC10482952

[48] Lee JM, et al. Cediranib and Olaparib combination compared with cediranib or Olaparib alone, or chemotherapy in Platinum-Resistant or primary Platinum-Refractory ovarian cancer: NRG-GY005. J Clin Oncol. 2024;42(36):4305–16. 39361946 10.1200/JCO.24.00683 PMC11652233

[49] Mirza MR, Matulonis UA. Niraparib in recurrent ovarian cancer. N Engl J Med. 2017;376(8):801–2. 28225676 10.1056/NEJMc1616633

[50] Moore KN, et al. Niraparib monotherapy for late-line treatment of ovarian cancer (QUADRA): a multicentre, open-label, single-arm, phase 2 trial. Lancet Oncol. 2019;20(5):636–48. 30948273 10.1016/S1470-2045(19)30029-4

[51] Monk BJ, et al. Niraparib first-line maintenance therapy in patients with newly diagnosed advanced ovarian cancer: final overall survival results from the PRIMA/ENGOT-OV26/GOG-3012 trial. Ann Oncol. 2024;35(11):981–92. 39284381 10.1016/j.annonc.2024.08.2241 PMC11934258

[52] Wu X, et al. Niraparib maintenance therapy using an individualised starting dose in patients with platinum-sensitive recurrent ovarian cancer (NORA): final overall survival analysis of a phase 3 randomised, placebo-controlled trial. EClinicalMedicine. 2024;72:102629. 38745967 10.1016/j.eclinm.2024.102629 PMC11090914

[53] Graybill WS, et al. Predictors of long-term progression-free survival in patients with ovarian cancer treated with niraparib in the PRIMA/ENGOT-OV26/GOG-3012 study. Int J Gynecol Cancer. 2024;34(7):1041–50. 38950925 10.1136/ijgc-2024-005356 PMC11228198

[54] Heitz F, et al. AGO-OVAR 28/ENGOT-ov57. Niraparib alone versus niraparib in combination with bevacizumab in patients with carboplatin-taxane-based chemotherapy in advanced ovarian cancer: a multicenter randomized phase III trial. Int J Gynecol Cancer. 2023;33(12):1966–9. 37935524 10.1136/ijgc-2023-004944

[55] Mirza MR, et al. Niraparib plus bevacizumab versus niraparib alone for platinum-sensitive recurrent ovarian cancer (NSGO-AVANOVA2/ENGOT-ov24): a randomised, phase 2, superiority trial. Lancet Oncol. 2019;20(10):1409–19. 31474354 10.1016/S1470-2045(19)30515-7

[56] Shi T, et al. Addendum: A phase II trial of cytoreductive surgery combined with niraparib maintenance in platinum-sensitive, secondary recurrent ovarian cancer: SGOG SOC-3 study. J Gynecol Oncol. 2022;33(4):e63. 35775688 10.3802/jgo.2022.33.e63 PMC9250854

[57] Gonzalez-Martin A, et al. Atezolizumab combined with platinum and maintenance niraparib for recurrent ovarian cancer with a platinum-Free interval > 6 months: ENGOT-OV41/GEICO 69-O/ANITA phase III trial. J Clin Oncol. 2024;42(36):4294–304. 39292975 10.1200/JCO.24.00668

[58] Hardesty MM, et al. OVARIO phase II trial of combination niraparib plus bevacizumab maintenance therapy in advanced ovarian cancer following first-line platinum-based chemotherapy with bevacizumab. Gynecol Oncol. 2022;166(2):219–29. 35690498 10.1016/j.ygyno.2022.05.020

[59] Liu G, et al. A novel combination of niraparib and anlotinib in platinum-resistant ovarian cancer: efficacy and safety results from the phase II, multi-center ANNIE study. EClinicalMedicine. 2022;54:101767. 36583171 10.1016/j.eclinm.2022.101767 PMC9793276

[60] Coleman RL, et al. Veliparib with First-Line chemotherapy and as maintenance therapy in ovarian cancer. N Engl J Med. 2019;381(25):2403–15. 31562800 10.1056/NEJMoa1909707 PMC6941439

[61] Swisher EM, et al. Impact of homologous recombination status and responses with veliparib combined with first-line chemotherapy in ovarian cancer in the phase 3 VELIA/GOG-3005 study. Gynecol Oncol. 2022;164(2):245–53. 34906376 10.1016/j.ygyno.2021.12.003

[62] Kristeleit R, et al. Rucaparib versus standard-of-care chemotherapy in patients with relapsed ovarian cancer and a deleterious BRCA1 or BRCA2 mutation (ARIEL4): an international, open-label, randomised, phase 3 trial. Lancet Oncol. 2022;23(4):465–78. 35298906 10.1016/S1470-2045(22)00122-X

[63] Andrew R, Clamp DL, et al. Rucaparib maintenance treatment for recurrent ovarian carcinoma: the effects of progression-free interval and prior therapies on efficacy and safety in the randomized phase 3 trial ARIEL3. Int J Gynecol Cancer. 2021;31(7):949–58. 34103386 10.1136/ijgc-2020-002240 PMC9445915

[64] Amit M, Oza AL, et al. Rucaparib versus chemotherapy for treatment of relapsed ovarian cancer with deleterious BRCA1 or BRCA2 mutation (ARIEL4): final results of an international, open-label, randomised, phase 3 trial. Lancet Oncol. 2025;26(2):249–64. 39914419 10.1016/S1470-2045(24)00674-0

[65] Li N, et al. Fuzuloparib maintenance therapy in patients with Platinum-Sensitive, recurrent ovarian carcinoma (FZOCUS-2): A multicenter, randomized, Double-Blind, Placebo-Controlled, phase III trial. J Clin Oncol. 2022;40(22):2436–46. 35404684 10.1200/JCO.21.01511

[66] Jing Nie HW, et al. Cost-effectiveness of Fuzuloparib compared to routine surveillance, niraparib and Olaparib for maintenance treatment of patients with germline BRCA1/2 mutation and platinum-sensitive recurrent ovarian carcinoma in China. Front Pharmacol. 2023;13:987337. 36686677 10.3389/fphar.2022.987337 PMC9846494

[67] Kong B, Zheng W. Mirvetuximab soravtansine: current and future applications. J Hematol Oncol. 2025;18(1):33. 40102896 10.1186/s13045-025-01686-2 PMC11921575

[68] Narayana R.V.L., Gupta, R. Exploring the therapeutic use and outcome of antibody-drug conjugates in ovarian cancer treatment. Oncogene. 2025;44(28):2343–2356. 10.1038/s41388-025-03448-3 PMC12229892 40514426

[69] Moore KN, et al. Phase III, randomized trial of Mirvetuximab Soravtansine versus chemotherapy in patients with platinum-resistant ovarian cancer: primary analysis of FORWARD I. Ann Oncol. 2021;32(6):757–65. 33667670 10.1016/j.annonc.2021.02.017

[70] Moore KN, et al. Mirvetuximab Soravtansine in FRalpha-Positive, Platinum-Resistant ovarian cancer. N Engl J Med. 2023;389(23):2162–74. 38055253 10.1056/NEJMoa2309169

[71] Gilbert L, et al. Safety and efficacy of Mirvetuximab soravtansine, a folate receptor alpha (FRalpha)-targeting antibody-drug conjugate (ADC), in combination with bevacizumab in patients with platinum-resistant ovarian cancer. Gynecol Oncol. 2023;170:241–7. 36736157 10.1016/j.ygyno.2023.01.020

[72] Gonzalez-Ochoa E, Veneziani AC, Oza AM. Mirvetuximab Soravtansine in Platinum-Resistant ovarian cancer. Clin Med Insights Oncol. 2023;17:11795549231187264. 37528890 10.1177/11795549231187264 PMC10387675

[73] Alvarez Secord A, et al. The efficacy and safety of Mirvetuximab Soravtansine in FRalpha-positive, third-line and later, recurrent platinum-sensitive ovarian cancer: the single-arm phase II PICCOLO trial. Ann Oncol. 2025;36(3):321–30. 39617145 10.1016/j.annonc.2024.11.011

[74] Van Gorp T, et al. Patient-reported outcomes from the MIRASOL trial evaluating Mirvetuximab Soravtansine versus chemotherapy in patients with folate receptor alpha-positive, platinum-resistant ovarian cancer: a randomised, open-label, phase 3 trial. Lancet Oncol. 2025;26(4):503–15. 40179908 10.1016/S1470-2045(25)00021-X

[75] O’Malley DM, et al. Maintenance with Mirvetuximab Soravtansine plus bevacizumab vs bevacizumab in FRalpha-high platinum-sensitive ovarian cancer. Future Oncol. 2024;20(32):2423–36. 39082675 10.1080/14796694.2024.2372241 PMC11520569

[76] Moore KN, et al. Safety and tolerability of Mirvetuximab Soravtansine monotherapy for folate receptor alpha-expressing recurrent ovarian cancer: an integrated safety summary. Gynecol Oncol. 2024;191:249–58. 39461270 10.1016/j.ygyno.2024.10.013

[77] Li X, et al. Discovery of STRO-002, a novel homogeneous ADC targeting folate receptor alpha, for the treatment of ovarian and endometrial cancers. Mol Cancer Ther. 2023;22(2):155–67. 36459691 10.1158/1535-7163.MCT-22-0322 PMC9890132

[78] Lee J-Y, Lorusso OA et al. D,., Efficacy and safety of luveltamab tazevibulin in patients with recurrent platinum-resistant ovarian cancer: results from the dose-optimization stage of the REFRαME-O1 (GOG-3086, ENGOT-79OV, and APGOT-OV9) phase 2/3 study. 2025 SGO Annual Meeting on Women’s Cancer, 2025;14(17): p. Seattle, WA. Abstract 922978.

[79] Call. JA, et al. A phase 1/2 study of Rinatabart Sesutecan (PRO1184), a novel folate receptor alpha-derected antibody-drug conjugate, in patients with locally advanced and/or metastatic solid tumors. J ImmunoTherapy Cancer. 2023;11(suppl1):A803.

[80] Angeles A, Secord et al. A phase 3, open-label, randomized study of Rinatabart Sesutecan (Rina-S) vs in- vestigator’s choice (IC) of chemotherapy in patients with platinum-resistant ovarian cancer (PROC). J Clin Oncol. 2025;43:suppl.TPS5627.

[81] McNamara B et al. Antibody-Drug conjugates (ADC) in HER2/neu-Positive gynecologic tumors. Molecules, 2023;28(21). 10.3390/molecules28217389 PMC10650896 37959808

[82] Meric-Bernstam F, et al. Efficacy and safety of trastuzumab Deruxtecan in patients with HER2-Expressing solid tumors: primary results from the DESTINY-PanTumor02 phase II trial. J Clin Oncol. 2024;42(1):47–58. 37870536 10.1200/JCO.23.02005 PMC10730032

[83] Mutlu L, et al. Trastuzumab Deruxtecan (DS-8201a), a HER2-targeting antibody-drug conjugate, demonstrates in vitro and in vivo antitumor activity against primary and metastatic ovarian tumors overexpressing HER2. Clin Exp Metastasis. 2024;41(5):765–75. 38909139 10.1007/s10585-024-10297-z

[84] Andrikopoulou A, et al. Real-world evidence of trastuzumab Deruxtecan (T-DXd) efficacy in HER2-expressing gynecological malignancies. BMC Cancer. 2024;24(1):1503. 39639215 10.1186/s12885-024-13226-1 PMC11622448

[85] Suzuki M, et al. Visualization of intratumor pharmacokinetics of [fam-] trastuzumab Deruxtecan (DS-8201a) in HER2 heterogeneous model using Phosphor-integrated Dots imaging analysis. Clin Cancer Res. 2021;27(14):3970–9. 33980613 10.1158/1078-0432.CCR-21-0397

[86] Gerber DE, et al. Phase Ia study of Anti-NaPi2b Antibody-Drug conjugate lifastuzumab Vedotin DNIB0600A in patients with Non-Small cell lung cancer and Platinum-Resistant ovarian cancer. Clin Cancer Res. 2020;26(2):364–72. 31540980 10.1158/1078-0432.CCR-18-3965

[87] Moore KN, et al. Phase 1b study of anti-NaPi2b antibody-drug conjugate lifastuzumab Vedotin (DNIB0600A) in patients with platinum-sensitive recurrent ovarian cancer. Gynecol Oncol. 2020;158(3):631–9. 32534811 10.1016/j.ygyno.2020.05.039

[88] Banerjee S, et al. Anti-NaPi2b antibody-drug conjugate lifastuzumab Vedotin (DNIB0600A) compared with pegylated liposomal doxorubicin in patients with platinum-resistant ovarian cancer in a randomized, open-label, phase II study. Ann Oncol. 2018;29(4):917–23. 29401246 10.1093/annonc/mdy023

[89] Richardson DL, Oaknin HE, Randall A, Banerjee LM, Taylor SN. Uplift (ENGOT-ov67): A pivotal cohort to evaluate XMT-1536 (upifitamab rilsodotin), a NaPi2b-directed antibody drug conjugate for platinum-resistant ovarian cancer. GYNECOLOGIC CANCER. 2021;39:15.

[90] Debra L, Richardson et al. UP-NEXT (GOG-3049/ENGOT-Ov71-NSGO-CTU): A study of Upitifamab Rilsodotin (UpRi), a NaPi2b-directed antibody drug conjugate (ADC), in platinum-sensitive recurrent ovarian cancer. GYNECOLOGIC CANCER, 2023;41:suppl TPS5614.

[91] Santin AD, et al. Safety and activity of anti-mesothelin antibody-drug conjugate anetumab Ravtansine in combination with pegylated-liposomal doxorubicin in platinum-resistant ovarian cancer: multicenter, phase Ib dose escalation and expansion study. Int J Gynecol Cancer. 2023;33(4):562–70. 36564099 10.1136/ijgc-2022-003927 PMC10086500

[92] Alqaisi HA, et al. Randomized phase II study of bevacizumab with weekly anetumab Ravtansine or weekly Paclitaxel in Platinum-Resistant/Refractory High-Grade ovarian cancer (NCI Trial). Clin Cancer Res. 2025;31(6):993–1001. 39836408 10.1158/1078-0432.CCR-24-3128 PMC11911801

[93] Weekes CD, et al. Phase I study of DMOT4039A, an Antibody-Drug conjugate targeting mesothelin, in patients with unresectable pancreatic or Platinum-Resistant ovarian cancer. Mol Cancer Ther. 2016;15(3):439–47. 26823490 10.1158/1535-7163.MCT-15-0693

[94] Hamilton E, et al. Tamrintamab Pamozirine (SC-003) in patients with platinum-resistant/refractory ovarian cancer: findings of a phase 1 study. Gynecol Oncol. 2020;158(3):640–5. 32513564 10.1016/j.ygyno.2020.05.038 PMC8227801

[95] de Bono JS, et al. Tisotumab Vedotin in patients with advanced or metastatic solid tumours (InnovaTV 201): a first-in-human, multicentre, phase 1–2 trial. Lancet Oncol. 2019;20(3):383–93. 30745090 10.1016/S1470-2045(18)30859-3

[96] Blank S, Tehrani HMO, Ghamande S, Jain S, Nicacio L, Soumaoro I, O’Malley DM. InnovaTV 208: new weekly dosing cohort in the phase II study of tisotumab Vedotin in platinum-resistant ovarian cancer. Ann Oncol. 2020;08:1021.

[97] Isabelle Laure Ray-Coquard. REJOICE-Ovarian01: A phase 2/3 study of Raludotatug Deruxtecan (R-DXd) in patients with platinum-resistant ovarian cancer (OVC). J Clin Oncol, 2024;42(16):suppl TPS5625.

[98] Suzuki H, et al. Raludotatug deruxtecan, a CDH6-Targeting Antibody-Drug conjugate with a DNA topoisomerase I inhibitor dxd, is efficacious in human ovarian and kidney cancer models. Mol Cancer Ther. 2024;23(3):257–71. 38205802 10.1158/1535-7163.MCT-23-0287 PMC10911705

[99] Jian A, et al. How to design next-generation of antibody-drug conjugates for cancer treatment: lessons from unsuccessful clinical trials. Cancer Lett. 2025;623:217535. 39924073 10.1016/j.canlet.2025.217535

[100] Chambers LM, Eskander RN, O’Malley DM. Targeting the future: Antibody-Drug conjugates (ADCs) in platinum-sensitive ovarian cancer in the post-PARP era. Ann Oncol. 2025;36(3):244–6. 39984223 10.1016/j.annonc.2025.01.015

[101] Zannoni GF et al. Folate receptor alpha in advanced epithelial ovarian cancer: diagnostic role and therapeutic implications of a clinically validated biomarker. Int J Mol Sci. 2025;26(11). 10.3390/ijms26115222 PMC12154392 40508029

[102] Zhu S, et al. Combination strategies to maximize the benefits of cancer immunotherapy. J Hematol Oncol. 2021;14(1):156. 34579759 10.1186/s13045-021-01164-5 PMC8475356

[103] Pardoll DM. The Blockade of immune checkpoints in cancer immunotherapy. Nat Rev Cancer. 2012;12(4):252–64. 22437870 10.1038/nrc3239 PMC4856023

[104] Colombo I et al. Chasing immune checkpoint inhibitors in ovarian cancer: novel combinations and biomarker discovery. Cancers (Basel). 2023;15(12). 10.3390/cancers15123220 PMC10296292 37370830

[105] Gupta R, et al. Immune evasion in ovarian cancer: implications for immunotherapy and emerging treatments. Trends Immunol. 2025;46(2):166–81. 39855990 10.1016/j.it.2024.12.006 PMC11835538

[106] Abiko K, et al. PD-L1 on tumor cells is induced in Ascites and promotes peritoneal dissemination of ovarian cancer through CTL dysfunction. Clin Cancer Res. 2013;19(6):1363–74. 23340297 10.1158/1078-0432.CCR-12-2199

[107] Morand S et al. Ovarian cancer immunotherapy and personalized medicine. Int J Mol Sci, 2021;22(12). 10.3390/ijms22126532 PMC8234871 34207103

[108] Disis ML, et al. Efficacy and safety of avelumab for patients with recurrent or refractory ovarian cancer: phase 1b results from the JAVELIN solid tumor trial. JAMA Oncol. 2019;5(3):393–401. 30676622 10.1001/jamaoncol.2018.6258 PMC6439837

[109] Matulonis UA, et al. Antitumor activity and safety of pembrolizumab in patients with advanced recurrent ovarian cancer: results from the phase II KEYNOTE-100 study. Ann Oncol. 2019;30(7):1080–7. 31046082 10.1093/annonc/mdz135

[110] Baas P, et al. First-line nivolumab plus ipilimumab in unresectable malignant pleural mesothelioma (CheckMate 743): a multicentre, randomised, open-label, phase 3 trial. Lancet. 2021;397(10272):375–86. 33485464 10.1016/S0140-6736(20)32714-8

[111] Mony U, Veeraraghavan VP. Outcomes of tumor-infiltrating lymphocyte therapy in solid tumours - A systematic review and meta analysis. Crit Rev Oncol Hematol. 2025;209:104671. 39978425 10.1016/j.critrevonc.2025.104671

[112] Zamarin D, et al. Randomized phase II trial of nivolumab versus nivolumab and ipilimumab for recurrent or persistent ovarian cancer: an NRG oncology study. J Clin Oncol. 2020;38(16):1814–23. 32275468 10.1200/JCO.19.02059 PMC7255977

[113] Zsiros E, et al. Efficacy and safety of pembrolizumab in combination with bevacizumab and oral metronomic cyclophosphamide in the treatment of recurrent ovarian cancer: A phase 2 nonrandomized clinical trial. JAMA Oncol. 2021;7(1):78–85. 33211063 10.1001/jamaoncol.2020.5945 PMC7677872

[114] Lampert EJ, et al. Combination of PARP inhibitor olaparib, and PD-L1 inhibitor durvalumab, in recurrent ovarian cancer: a Proof-of-Concept phase II study. Clin Cancer Res. 2020;26(16):4268–79. 32398324 10.1158/1078-0432.CCR-20-0056 PMC7442720

[115] Konstantinopoulos PA, et al. Single-Arm phases 1 and 2 trial of niraparib in combination with pembrolizumab in patients with recurrent Platinum-Resistant ovarian carcinoma. JAMA Oncol. 2019;5(8):1141–9. 31194228 10.1001/jamaoncol.2019.1048 PMC6567832

[116] Moore KN, et al. Atezolizumab, bevacizumab, and chemotherapy for newly diagnosed stage III or IV ovarian cancer: Placebo-Controlled randomized phase III trial (IMagyn050/GOG 3015/ENGOT-OV39). J Clin Oncol. 2021;39(17):1842–55. 33891472 10.1200/JCO.21.00306 PMC8189598

[117] Pujade-Lauraine E, et al. Avelumab alone or in combination with chemotherapy versus chemotherapy alone in platinum-resistant or platinum-refractory ovarian cancer (JAVELIN ovarian 200): an open-label, three-arm, randomised, phase 3 study. Lancet Oncol. 2021;22(7):1034–46. 34143970 10.1016/S1470-2045(21)00216-3

[118] Ledermann JA, et al. Molecular determinants of clinical outcomes of pembrolizumab in recurrent ovarian cancer: exploratory analysis of KEYNOTE-100. Gynecol Oncol. 2023;178:119–29. 37862791 10.1016/j.ygyno.2023.09.012

[119] Mateiou C, et al. Spatial tumor immune microenvironment phenotypes in ovarian cancer. NPJ Precis Oncol. 2024;8(1):148. 39026018 10.1038/s41698-024-00640-8 PMC11258306

[120] Liu P, et al. Combined PD-1/PD-L1 and tumor-infiltrating immune cells redefined a unique molecular subtype of high-grade serous ovarian carcinoma. BMC Genomics. 2022;23(1):51. 35026984 10.1186/s12864-021-08265-y PMC8759258

[121] Choucair K, et al. TMB: a promising immune-response biomarker, and potential spearhead in advancing targeted therapy trials. Cancer Gene Ther. 2020;27(12):841–53. 32341410 10.1038/s41417-020-0174-y

[122] Marabelle A, et al. Association of tumour mutational burden with outcomes in patients with advanced solid tumours treated with pembrolizumab: prospective biomarker analysis of the multicohort, open-label, phase 2 KEYNOTE-158 study. Lancet Oncol. 2020;21(10):1353–65. 32919526 10.1016/S1470-2045(20)30445-9

[123] Chalmers ZR, et al. Analysis of 100,000 human cancer genomes reveals the landscape of tumor mutational burden. Genome Med. 2017;9(1):34. 28420421 10.1186/s13073-017-0424-2 PMC5395719

[124] Landen CN, et al. Influence of genomic landscape on cancer immunotherapy for newly diagnosed ovarian cancer: biomarker analyses from the IMagyn050 randomized clinical trial. Clin Cancer Res. 2023;29(9):1698–707. 36595569 10.1158/1078-0432.CCR-22-2032 PMC10150250

[125] Fares CM, et al. Homologous recombination deficiency and molecular subtype are associated with immunogenicity in ovarian cancer. Biomark Med. 2022;16(10):771–82. 35642517 10.2217/bmm-2022-0044

[126] Xie N, et al. Neoantigens: promising targets for cancer therapy. Signal Transduct Target Ther. 2023;8(1):9. 36604431 10.1038/s41392-022-01270-x PMC9816309

[127] Silva SB, Wanderley CWS, Colli LM. Immune checkpoint inhibitors in tumors harboring homologous recombination deficiency: challenges in attaining efficacy. Front Immunol. 2022;13:826577. 35211121 10.3389/fimmu.2022.826577 PMC8860897

[128] Vinayak S, et al. Open-label clinical trial of niraparib combined with pembrolizumab for treatment of advanced or metastatic Triple-Negative breast cancer. JAMA Oncol. 2019;5(8):1132–40. 31194225 10.1001/jamaoncol.2019.1029 PMC6567845

[129] Varghese A, et al. Tertiary lymphoid structures: exploring opportunities to improve immunotherapy in ovarian cancer. Front Immunol. 2025;16:1473969. 40475770 10.3389/fimmu.2025.1473969 PMC12137288

[130] Liu J, et al. Construction of an immune cell infiltration score to evaluate the prognosis and therapeutic efficacy of ovarian cancer patients. Front Immunol. 2021;12:751594. 34745124 10.3389/fimmu.2021.751594 PMC8564196

[131] Gao W, et al. Identification of three subtypes of ovarian cancer and construction of prognostic models based on immune-related genes. J Ovarian Res. 2024;17(1):208. 39434163 10.1186/s13048-024-01526-w PMC11492668

[132] Shah SM, et al. Exploring Co-occurring POLE exonuclease and Non-exonuclease domain mutations and their impact on tumor mutagenicity. Cancer Res Commun. 2024;4(1):213–25. 38282550 10.1158/2767-9764.CRC-23-0312 PMC10812383

[133] Kim SI, et al. Durvalumab with or without Tremelimumab plus chemotherapy in HRR non-mutated, platinum-resistant ovarian cancer (KGOG 3045): A phase II umbrella trial. Gynecol Oncol. 2024;182:7–14. 38246047 10.1016/j.ygyno.2023.12.029

[134] Liu T, et al. The role of interferons in ovarian cancer progression: hinderer or promoter? Front Immunol. 2022;13:1087620. 36618371 10.3389/fimmu.2022.1087620 PMC9810991

[135] Abiko K, et al. Dynamic host immunity and PD-L1/PD-1 Blockade efficacy: developments after IFN-gamma from lymphocytes induces PD-L1 expression and promotes progression of ovarian cancer. Br J Cancer. 2023;128(3):461–7. 36068276 10.1038/s41416-022-01960-x PMC9938281

[136] Reddy SU et al. IFNgamma-Induced Bcl3, PD-L1 and IL-8 signaling in ovarian cancer: mechanisms and clinical significance. Cancers (Basel). 2024;16(15). 10.3390/cancers16152676 PMC11311860 39123403

[137] Maas RJA, et al. Increased peritoneal TGF-beta1 is associated with ascites-induced NK-cell dysfunction and reduced survival in high-grade epithelial ovarian cancer. Front Immunol. 2024;15:1448041. 39376560 10.3389/fimmu.2024.1448041 PMC11456434

[138] Tang M, et al. Cross-talk between ovarian cancer cells and macrophages through Periostin promotes macrophage recruitment. Cancer Sci. 2018;109(5):1309–18. 29527764 10.1111/cas.13567 PMC5980394

[139] Okla K. Myeloid-Derived suppressor cells (MDSCs) in ovarian Cancer-Looking back and forward. Cells, 2023;12(14). 10.3390/cells12141912 PMC10377883 37508575

[140] Gourdin N, et al. Autocrine adenosine regulates tumor polyfunctional CD73(+)CD4(+) effector T cells devoid of immune checkpoints. Cancer Res. 2018;78(13):3604–18. 29559470 10.1158/0008-5472.CAN-17-2405

[141] Bareche Y et al. High-dimensional analysis of the adenosine pathway in high-grade serous ovarian cancer. J Immunother Cancer. 2021;9(3). 10.1136/jitc-2020-001965 PMC7996652 33771891

[142] Ghorani E, Swanton C, Quezada SA. Cancer cell-intrinsic mechanisms driving acquired immune tolerance. Immunity. 2023;56(10):2270–95. 37820584 10.1016/j.immuni.2023.09.004

[143] Jimenez-Sanchez A, et al. Unraveling tumor-immune heterogeneity in advanced ovarian cancer uncovers Immunogenic effect of chemotherapy. Nat Genet. 2020;52(6):582–93. 32483290 10.1038/s41588-020-0630-5 PMC8353209

[144] Chap BS, et al. Crosstalk of T cells within the ovarian cancer microenvironment. Trends Cancer. 2024;10(12):1116–30. 39341696 10.1016/j.trecan.2024.09.001

[145] Cai L, et al. Correction: Targeting LAG-3, TIM-3, and TIGIT for cancer immunotherapy. J Hematol Oncol. 2023;16(1):105. 37773132 10.1186/s13045-023-01503-8 PMC10543833

[146] Lu C, Tan Y. Promising immunotherapy targets: TIM3, LAG3, and TIGIT joined the party. Mol Ther Oncol. 2024;32(1):200773. 38596295 10.1016/j.omton.2024.200773 PMC10905042

[147] Pignata S, et al. Overall survival and patient-reported outcome results from the placebo-controlled randomized phase III IMagyn050/GOG 3015/ENGOT-OV39 trial of Atezolizumab for newly diagnosed stage III/IV ovarian cancer. Gynecol Oncol. 2023;177:20–31. 37625235 10.1016/j.ygyno.2023.06.018 PMC10986425

[148] Gaitskell K, et al. Angiogenesis inhibitors for the treatment of epithelial ovarian cancer. Cochrane Database Syst Rev. 2023;4(4):CD007930. 37185961 10.1002/14651858.CD007930.pub3 PMC10111509

[149] Fumet JD, et al. Precision medicine phase II study evaluating the efficacy of a double immunotherapy by durvalumab and Tremelimumab combined with Olaparib in patients with solid cancers and carriers of homologous recombination repair genes mutation in response or stable after Olaparib treatment. BMC Cancer. 2020;20(1):748. 32778095 10.1186/s12885-020-07253-x PMC7418426

[150] Awada A, et al. Immunotherapy in the treatment of Platinum-Resistant ovarian cancer: current perspectives. Onco Targets Ther. 2022;15:853–66. 35982728 10.2147/OTT.S335936 PMC9379118

[151] Domchek SM, et al. Olaparib and durvalumab in patients with germline BRCA-mutated metastatic breast cancer (MEDIOLA): an open-label, multicentre, phase 1/2, basket study. Lancet Oncol. 2020;21(9):1155–64. 32771088 10.1016/S1470-2045(20)30324-7

[152] Hardy-Bessard, A.C, et al. Dostarlimab and niraparib in primary advanced ovarian cancer. Ann Oncol. 2025;S0923-7534(25)00201–7. 10.1016/j.annonc.2025.05.009 10.1016/j.annonc.2025.05.009 40461381

[153] Gonzalez-Martin A, et al. Lenvatinib plus pembrolizumab for patients with previously treated advanced ovarian cancer: results from the phase 2 multicohort LEAP-005 study. Gynecol Oncol. 2024;186:182–90. 38718741 10.1016/j.ygyno.2024.04.011

[154] Calo CA, et al. Combination lenvatinib plus pembrolizumab in the treatment of ovarian clear cell carcinoma: A case series. Gynecol Oncol Rep. 2023;46:101171. 37065539 10.1016/j.gore.2023.101171 PMC10090985

[155] Chen J, et al. Anti-mesothelin CAR-T immunotherapy in patients with ovarian cancer. Cancer Immunol Immunother. 2023;72(2):409–25. 35925286 10.1007/s00262-022-03238-w PMC10991348

[156] Lorentzen CL, et al. Clinical advances and ongoing trials on mRNA vaccines for cancer treatment. Lancet Oncol. 2022;23(10):e450–8. 36174631 10.1016/S1470-2045(22)00372-2 PMC9512276

[157] Chen Q, Sun Y, Li H. Application of CAR-T cell therapy targeting mesothelin in solid tumor treatment. Discov Oncol. 2024;15(1):289. 39023820 10.1007/s12672-024-01159-x PMC11258118

[158] Xu T, et al. Efficacy and safety of novel multiple-chain DAP-CAR-T cells targeting mesothelin in ovarian cancer and mesothelioma: a single-arm, open-label and first-in-human study. Genome Med. 2024;16(1):133. 39548510 10.1186/s13073-024-01405-5 PMC11568615

[159] Hassan R, et al. Mesothelin-targeting T cell receptor fusion construct cell therapy in refractory solid tumors: phase 1/2 trial interim results. Nat Med. 2023;29(8):2099–109. 37501016 10.1038/s41591-023-02452-y PMC10427427

[160] Cutri-French C et al. CAR-T cell therapy in ovarian cancer: where are we now? Diagnostics (Basel). 2024;14(8). 10.3390/diagnostics14080819 PMC11049291 38667465

[161] Borges F, et al. Trial watch: anticancer vaccination with dendritic cells. Oncoimmunology. 2024;13(1):2412876. 39398476 10.1080/2162402X.2024.2412876 PMC11469433

[162] Holloway RW, et al. A phase III, multicenter, randomized study of olvimulogene Nanivacirepvec followed by platinum-doublet chemotherapy and bevacizumab compared with platinum-doublet chemotherapy and bevacizumab in women with platinum-resistant/refractory ovarian cancer. Int J Gynecol Cancer. 2023;33(9):1458–63. 37666539 10.1136/ijgc-2023-004812

[163] Moufarrij S, O’Cearbhaill RE. Novel therapeutics in ovarian cancer: expanding the toolbox. Curr Oncol. 2023;31(1):97–114. 38248092 10.3390/curroncol31010007 PMC10814452

[164] Block MS, et al. The oncolytic adenovirus TILT-123 with pembrolizumab in platinum resistant or refractory ovarian cancer: the phase 1a PROTA trial. Nat Commun. 2025;16(1):1381. 39910037 10.1038/s41467-025-56482-w PMC11799410

[165] Vaishampayan UN et al. Nemvaleukin alfa, a modified interleukin-2 cytokine, as monotherapy and with pembrolizumab in patients with advanced solid tumors (ARTISTRY-1). J Immunother Cancer. 2024;12(11). 10.1136/jitc-2024-010143 PMC11580269 39567211

[166] Rachel Grisham et al. GOG-3097/ENGOT-ov81/GTG-UK/RAMP 301: a phase 3, randomized trial evaluating avutometinib plus defactinib compared with investigator’s choice of treatment in patients with recurrent low grade serous ovarian cancer. Int J Gynecol Cancer. 2025;2024–005919. 10.1136/ijgc-2024-005919 10.1136/ijgc-2024-005919 39375168

[167] Blair, McNamara, et al. Preclinical efficacy of RAF/MEK clamp Avutometinib in combination with FAK Inhibition in low grade serous ovarian cancer. Gynecol Oncol. 2024;183:133–40. 38493021 10.1016/j.ygyno.2024.01.028

[168] Rachel Grisham BM et al. A phase III, randomized trial evaluating avutometinib plus defactinib compared with investigator’s choice of therapy among patients with recurrent low-grade serous ovarian cancer: GOG- 3097/ENGOT-OV81/NCRI/RAMP 301. 2024;190:s282–3. 10.1136/ijgc-2024-005919 39375168

[169] Chereau E, et al. Comparison of peritoneal carcinomatosis scoring methods in predicting resectability and prognosis in advanced ovarian cancer. Am J Obstet Gynecol. 2010;202(2):e1781–17810. 10.1016/j.ajog.2009.10.856 20113693

[170] Feng Z, et al. A triage strategy in advanced ovarian cancer management based on multiple predictive models for R0 resection: a prospective cohort study. J Gynecol Oncol. 2018;29(5):e65. 30022629 10.3802/jgo.2018.29.e65 PMC6078898

[171] Pujade-Lauraine E, et al. Maintenance Olaparib Rechallenge in patients with platinum-sensitive relapsed ovarian cancer previously treated with a PARP inhibitor (OReO/ENGOT-ov38): a phase IIIb trial. Ann Oncol. 2023;34(12):1152–64. 37797734 10.1016/j.annonc.2023.09.3110

[172] Poveda A, et al. Olaparib maintenance monotherapy in platinum-sensitive relapsed ovarian cancer patients without a germline BRCA1/BRCA2 mutation: OPINION primary analysis. Gynecol Oncol. 2022;164(3):498–504. 35063276 10.1016/j.ygyno.2021.12.025

[173] Lorusso D, et al. Updated progression-free survival and final overall survival with maintenance Olaparib plus bevacizumab according to clinical risk in patients with newly diagnosed advanced ovarian cancer in the phase III PAOLA-1/ENGOT-ov25 trial. Int J Gynecol Cancer. 2024;34(4):550–8. 38129136 10.1136/ijgc-2023-004995 PMC10982633

[174] Pignata S, et al. Maintenance Olaparib in patients with platinum-sensitive relapsed ovarian cancer: outcomes by somatic and germline BRCA and other homologous recombination repair gene mutation status in the ORZORA trial. Gynecol Oncol. 2023;172:121–9. 37030280 10.1016/j.ygyno.2023.03.019

[175] Coleman RL, et al. Real-world overall survival in second-line maintenance niraparib monotherapy versus active surveillance in patients with BRCA wild-type recurrent ovarian cancer. Ther Adv Med Oncol. 2024;16:17588359241292272. 39552638 10.1177/17588359241292272 PMC11565610

[176] O’Malley DM, et al. PARP inhibitors in ovarian cancer: A review. Target Oncol. 2023;18(4):471–503. 37268756 10.1007/s11523-023-00970-w PMC10344972

